# TENG-Boosted Smart Sports with Energy Autonomy and Digital Intelligence

**DOI:** 10.1007/s40820-025-01778-1

**Published:** 2025-05-21

**Authors:** Yunlu Wang, Zihao Gao, Wei Wu, Yao Xiong, Jianjun Luo, Qijun Sun, Yupeng Mao, Zhong Lin Wang

**Affiliations:** 1 Physical Education Department, Northeastern University, Shenyang, 110819 People’s Republic of China; 2 Beijing Institute of Nanoenergy and Nanosystems, Chinese Academy of Sciences, Beijing, 101400 People’s Republic of China; 3 Shandong Zhongke Naneng Energy Technology Co., Ltd, Dongying, 257061 People’s Republic of China; 4 School of Strength and Conditioning Training, Beijing Sport University, Beijing, 100084 People’s Republic of China

**Keywords:** Triboelectric nanogenerator, Intelligent sports, Self-powered sensing, Physiological monitoring, Sports injury prevention

## Abstract

Technological advancements have profoundly transformed the sports domain, ushering it into the digital era. Services leveraging big data in intelligent sports—encompassing performance analytics, training statistical evaluations and metrics—have become indispensable. These tools are vital in aiding athletes with their daily training regimens and in devising sophisticated competition strategies, proving crucial in the pursuit of victory. Despite their potential, wearable electronic devices used for motion monitoring are subject to several limitations, including prohibitive cost, extensive energy usage, incompatibility with individual spatial structures, and flawed data analysis methodologies. Triboelectric nanogenerators (TENGs) have become instrumental in the development of self-powered devices/systems owing to their remarkable capacity to harnessing ambient high-entropy energy from the environment. This paper provides a thorough review of the advancements and emerging trends in TENG-based intelligent sports, focusing on physiological data monitoring, sports training performance, event refereeing assistance, and sports injury prevention and rehabilitation. Excluding the potential influence of sports psychological factors, this review provides a detailed discourse on present challenges and prospects for boosting smart sports with energy autonomy and digital intelligence. This study presents innovative insights and motivations for propelling the evolution of intelligent sports toward a more sustainable and efficient future for humanity.

## Highlights

- The recent advancements in triboelectric nanogenerator (TENG)-based sports equipment for smart sports are comprehensively reviewed.
- Thorough explorations of combining TENG technology and artificial intelligence/machine learning techniques to enhance smart sports are examined in this study.
- Comprehensive discussions on the opportunities and challenges of TENG-based smart sports are summarized.

## Graphical abstract

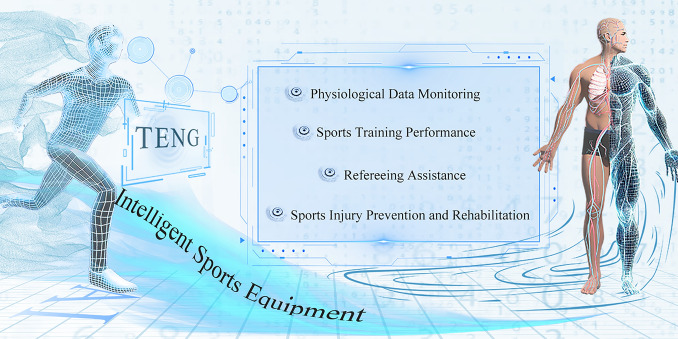

## Introduction

The benefits of exercise can significantly influence systemic health of humans, reaching far beyond their effect on individual organ systems [1]. Regular physical activity is a cornerstone of health promotion, providing benefits across various interconnected physiological systems such as cognitive, immune, neurohormonal, cardiovascular, and musculoskeletal systems. The multiorgan benefits after regular exercise (e.g., improved cardiorespiratory fitness) have been demonstrated to mitigate a broad spectrum of human diseases like cancer, cardiometabolic disorders, and frailty, and promote longevity [2–6]. The crucial role of physical activity for health and well-being is well acknowledged, with a directly proportional relationship observed between physical activity and health. Research suggests that individuals who aim to lead active, healthy lifestyles tend to enjoy longer and healthier lives [7]. As modern society and the economy rapidly evolve in the modern era, the significance of real-time and distributed sensing technology coupled with big data collection/analysis becomes increasingly pronounced in the Internet of Things (IoT) era [8]. The sports industry has experienced revolutionary technological changes, propelling it toward a truly digital, interactive, and intelligent era [9]. This field of research encompasses health data, training statistics, kinesiological analysis, competition support, safety forecasting, and information sharing. Exercise data not only enhance our understanding of human health and performance but also aid in decision-making [10–12]. It has become an indispensable tool for athletes, enabling them not just gather relevant data such as time, frequency, and speed of movement, but also to visualize their physical condition [13, 14].

The real-time tracking and monitoring of motion status typically rely on the use of widely distributed sensors [15]. However, current technologies fall short in meeting these objectives due to their lack of flexibility and portability. These technologies also often fail to exhibit adequate durability and frequently overlook environmental contamination concerns during motion [16]. Additionally, wearable electronic devices used for motion tracking face several limitations including high cost, substantial power consumption, inflexibility inadaptable to personalized structures, and flawed data analysis methods. Traditional sensors require an external power source, which is unsuitable for large-scale applications due to scalability issues, complex cabling, degradation, short lifespan, and high cost of battery replacement [17–19]. As society becomes increasingly interconnected and nonlinear by comprising a multitude of devices and energy sources, there is an urgent need for a portable, pervasive, and self-sustaining power supply. Sensors cannot feasibly power the IoT unless they are capable of harvesting energy from their surrounding environment to ensure long-term operation. Consequently, the development of an environmentally friendly, maintenance-free, and sustainable sensing technology is crucial to meet the demands of intended applications.

A range of energy harvesting technologies, such as thermoelectric generators, biofuel cells, solar cells, and piezoelectric nanogenerators, have been suggested for various wearable and portable applications [20]. These emerging self-powered devices can effectively mitigate the disadvantages associated with the use of batteries in conventional electronics. A novel low-frequency, high-entropy energy harvesting technology, triboelectric nanogenerator (TENG), is particularly well suited to harvest mechanical energy from both large motions (e.g., wind and water waves) and small, random energy sources (e.g., mechanical energy generated by human activities) [21]. TENG generates electricity by converting mechanical energy, utilizing the combined effects of triboelectricity and electrostatic induction. A separation or approach of two electrically conductive materials leads to a corresponding divergence or convergence of their electron clouds at the interface. When these electron clouds converge, the probability of electrons undergoing a jump decreases due to the reduction in potential barrier. Upon contact, electrons are transferred from the low-electron-affinity material with lower electron affinity to that with higher electron affinity, creating a potential difference that can be harnessed through an external circuit [22]. The operation process of TENGs can be categorized into five distinct modes: contact-separation mode, sliding mode, single-electrode mode, free-standing mode, and rolling mode, which enable the capture of subtle biomechanical motions across various planes [23]. Moreover, the lightweight nature, high flexibility, biocompatibility, and diverse selection of triboelectric materials of TENG devices make them ideal for integration into self-powered systems and be identified as promising power sources for the IoT [24–29].

A variety of self-powered devices based on TENG have been demonstrated for diverse applications, such as health/biomechanical/environmental monitoring, distributed energy harvesting, and intelligent transportation. TENGs offer significant advantages over other wearable devices such as sensors for monitoring human movement; their light weight minimizes obstruction to the human body during movement. Additionally, their high mechanical strength enables them to withstand multiplanar deformation, thereby extending device lifespan. Furthermore, TENGs can effectively monitor movement signals in extreme environments and perform real-time analysis, potentially advancing sports toward more environmentally friendly and intelligent practices. Data collection and analysis are fundamental processes in intelligent sports for managing and processing the triboelectric sensing signals derived from TENG monitoring of specific biochemical or physiological activities [30, 31].

**Fig. 1:**
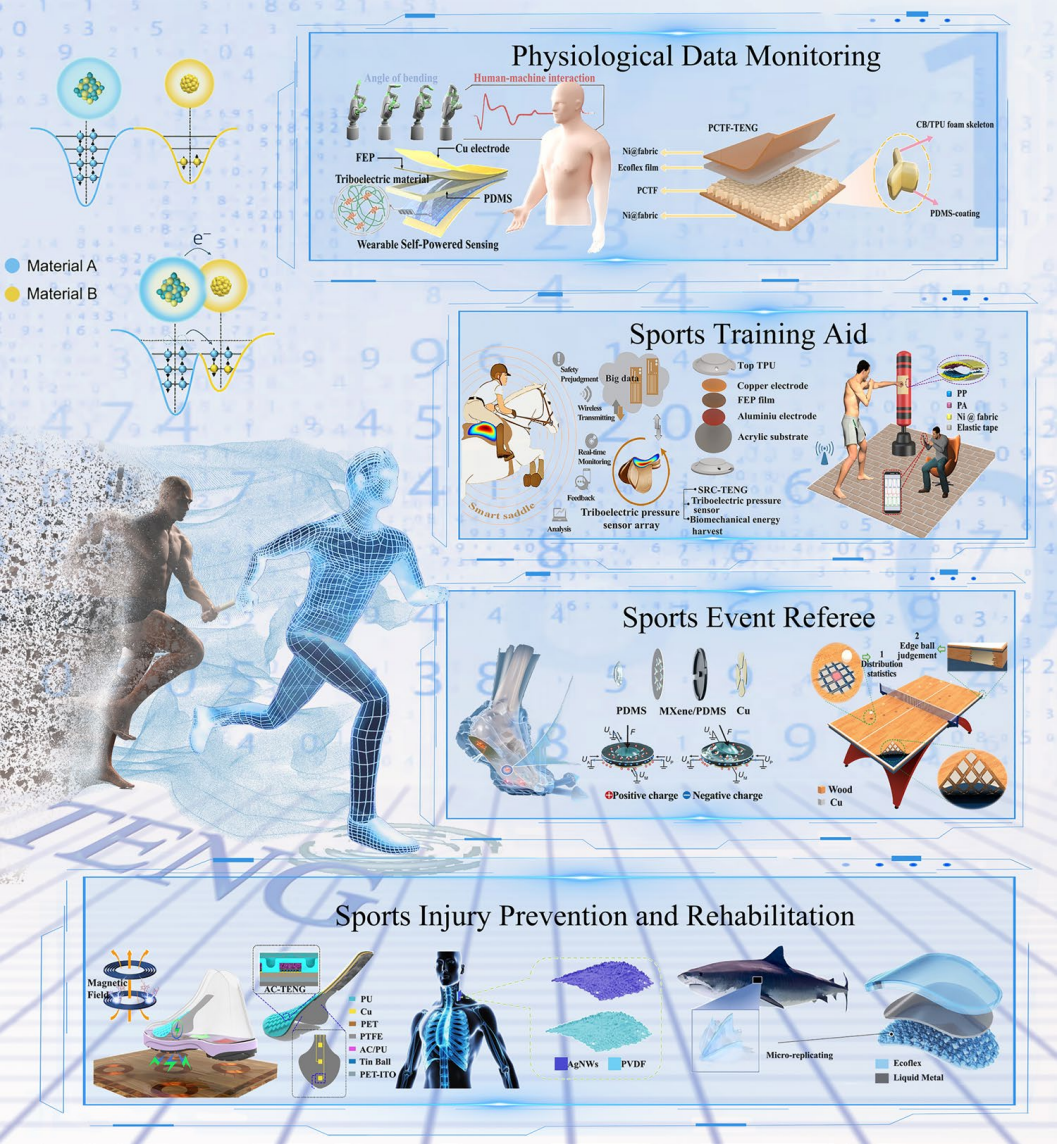
TENG-based intelligent sports equipment for self-powered monitoring, training, refereeing, and injury prevention. Reproduced with permission [41]. Copyright 2024, American Chemical Society. Reproduced with permission [43]. Copyright 2024, Elsevier. Reproduced with permission [60]. Copyright 2019, Elsevier. Reproduced with permission [65]. Copyright 2022, American Chemical Society. Reproduced with permission [77]. Copyright 2019, The Author(s). Reproduced with permission [79]. Copyright 2024, Wiley‐VCH GmbH. Reproduced with permission [96]. Copyright 2024, Elsevier. Reproduced with permission [109]. Copyright 2022, American Chemical Society. Reproduced with permission [110]. Copyright 2022, Elsevier

The use of wearable electronic sensors has significantly increased in sports science, providing athletes with essential physiological information to ensure optimal competitive performance [32]. However, the evaluation criteria for an athlete’s competitive status of athletes are multifaceted, considering various elements such as physiology, psychology, technology, and overall competitive performance. This paper provides a comprehensive overview of the evaluation and development trends of TENG-based intelligent sports equipment, setting aside the influence of sports psychological factors. The focus areas include physiological data monitoring, sports training performance, refereeing assistance, and sports injury prevention and rehabilitation, as shown in Fig. 1. Additionally, this paper summarizes and forecasts the existing challenges in the realm of smart sports for energy autonomy and digital intelligence. This will offer fresh perspectives to foster the progression of sports development toward environmentally friendly and intelligent growth.

## Intelligent Sports Technology for Self-Powered Motion Monitoring

With the advancement of technology, smart wearables are now capable of not only continuously monitoring individuals’ health, but also tracking their physical activity to facilitate better management of their well-being. In the realm of sports, the surveillance of athletes can enhance the beneficial effects of training such as improved health, preparation, and performance, while simultaneously reducing detrimental impacts, including overexertion, injury, and disease. Currently, image processing is a dominant technique for monitoring body movements, requiring significant image datasets for subsequent analysis [33]. However, video-assisted motion-capture systems require specific lighting conditions, and camera placement can greatly influence recognition accuracy. Furthermore, capturing a vast number of images may compromise user privacy. The inertial measurement unit (IMU) is another prevalent technology that amalgamates accelerometers and gyroscopes to estimate spatial movements [34]. Although IMUs are highly accurate and reliable, they generate vast amounts of data, exhibit cumulative error, and necessitate complex data analysis. Moreover, IMUs typically perform singular functions, with the integration of multiple single-function sensors being the current method for achieving multifunctionality. The considerable cost of these technologies is a significant impediment to their widespread adoption in practical applications.

The IoT necessitates the requirement for motion-sensing networks that can function continuously for extended periods without any maintenance [35]. The emergence of self-powered sensors, capable of directly converting detection information into electrical signals without the need for external power sources, presents a promising solution [36]. TENG represents an emerging electromechanical energy conversion platform technology and a self-powered sensing terminal, demonstrating significant application potential in the sports field. Compared to traditional sensors, TENG sensors offer distinct advantages in overall performance. A systematic comparison of different types of sensors across various aspects—including application, sensitivity, durability, and response/recovery times (as summarized in Table 1)—reveals that while all sensor types exhibit excellent sensitivity within specific pressure ranges and can accurately capture subtle human movements, the unique demands of sports (e.g., high-intensity impacts, prolonged continuous activity, and large-scale movements) impose more stringent requirements on durability, environmental adaptability, and long-term wearability. Furthermore, the unpredictability and dynamic variability of competitive sports demand superior sensing sensitivity, rapid response capabilities, and outstanding durability to enable real-time tracking of environmental conditions and subtle physiological changes in athletes. Owing to its higher energy conversion efficiency, broader operating pressure range, superior environmental adaptability, and lower manufacturing cost, TENG sensors exhibit unique suitability for sports applications. These advantages have made TENG sensors a research focus, leading to extensive studies across various sports disciplines, with a depth and breadth of research and application surpassing that of other sensor technologies.

**Table 1:** Performance comparison of TENGs and other energy harvesting technologies for sports applications

Type	Application	Sensitivity	Durability	Response/ Recovery
Piezoelectric sensors	Fingers bending angle, walking and running [201]	0.9 kPa^−1^ (0–1 kPa)	10,000 times	165 ms/132 ms
	Volleyball hitting gesture recognition and elbow joint state detection [202]		20 min	
	The pressure between the volleyball and glove, volleyball match refereeing [203]	Soft sensors: 0.056 V N^–1^ Hard sensors: 0.032 V N^–1^		
	Rectify wrong exertion or posture of playing badminton, prevent chronic sports injuries [204]	2.8 V kPa^–1^ (3.4–22.2 kPa)	7000 times	4 ms/-
	Joint changes and sliding trajectories in speed skating [205]		2000s	43 ms/-
Thermoelectric sensors	Bending states of the fingers, wrists, elbows and knees [206]	GF = 0.88 (0–40%) GF = 1.08 (40–100%)	1000 times (500% stretching)	< 1500 ms/-
	Shooting angle of basketball [207]	GF = 2.7 (10–40%) GF = 3.6 (40–50%)	1200 times (30% stretching)	≈500 ms/-
Capacitive sensors	Monitoring different movement states such as walking, running and jumping [208]	GF = 6.46 (0–140%)	3300 times (10% stretching)	134 ms/166 ms
	Monitoring respiration, pulse and elbow Movement postures in basketball game [209]	33.5 kPa^−1^ (0–200 Pa)	100,000 times	27 ms/52 ms
	Sweat monitoring [210]	6.021 pF nL^−1^		
Electromagnetic sensors	Monitor different running speeds [211]	0.59 mV µm^−1^ (1.7 kHz)	2000 times	
	Visually impaired people running on the track [212]	5–10 mV		32 ms/-
Triboelectric sensors	Pressure sensing of bicycle seats [213]		10,800 times/6 h	80 ms/52 ms
	Distinguish the walking, running, jumping, leaping and limping [214]	S = 1.76 kPa^−1^ (< 16 kPa)	100,000 times	50 ms/76 ms
	Distinguish different exercise rehabilitation movements [215]	14 mV kPa^−1^ (0–50 kPa)	5000 times (15 kPa)	161 ms/160 ms
		0.35 mV kPa^−1^ (50–380 kPa)		
	Monitoring table tennis ball speed, landing point and edge balls [77]	0.78 V ms^−1^ (ball speed < 4.5 ms^−1^) 0.21 V ms^−1^ (ball speed > 4.5 ms^−1^)	20,000 times	< 25 ms/-

**Fig. 2:**
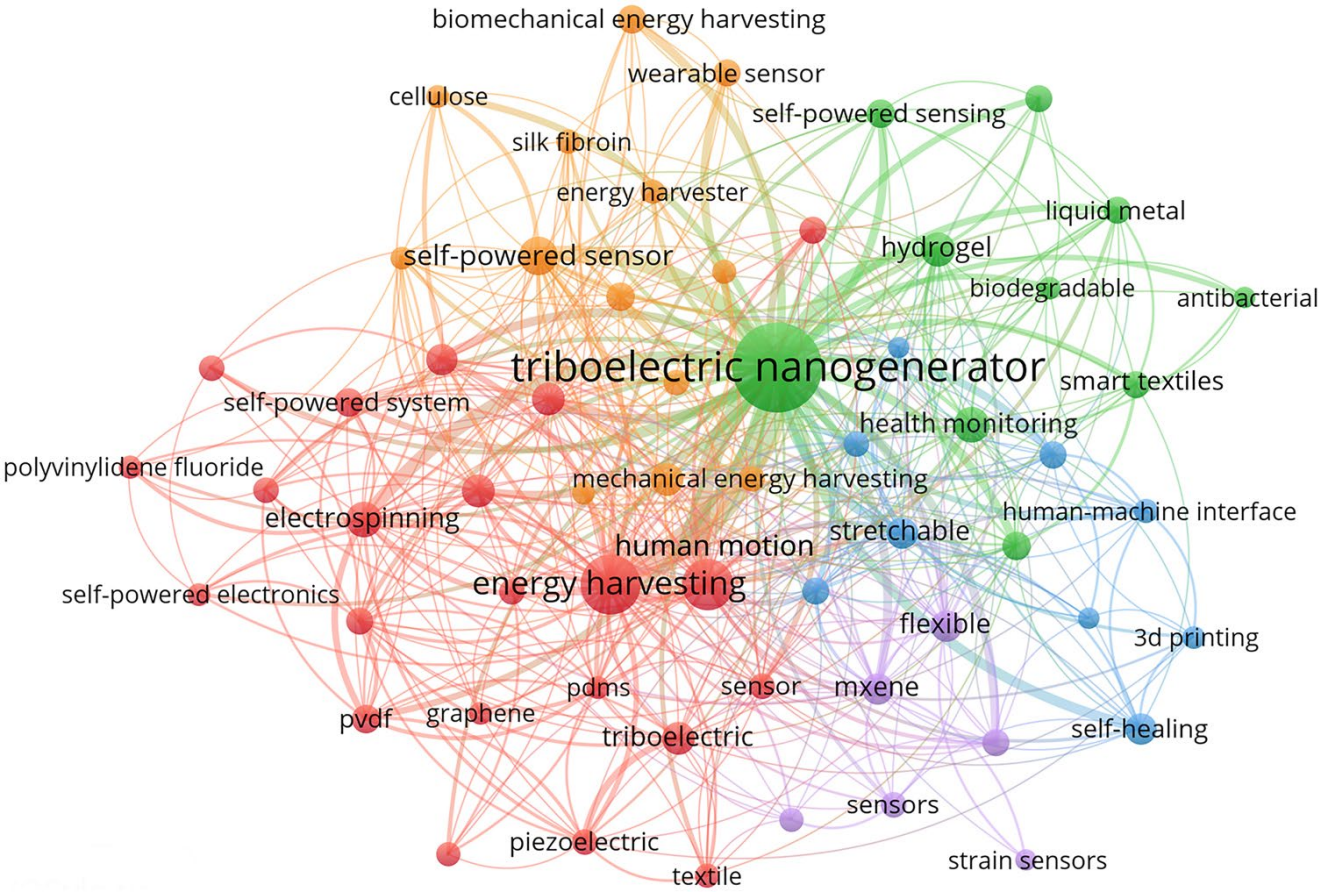
Keywords co-occurrence network graph of TENG-related publications

The co-occurrence network graph of keywords from TENG-related publications (Fig. 2) illustrates that alongside energy harvesting for self-powered smart devices, wearables based on TENG technology for human health monitoring and human motion tracking are also gaining significant attention. However, human sport performance is subject to a complex interplay of multifactorial determinants, including individual physiological characteristics, environmental conditions, external variables, and psychological parameters. Psychological influences arise from the dynamic interaction between intrinsic determinants (e.g., motivational states, anxiety levels, self-efficacy, and attentional focus) and extrinsic environmental/social factors, collectively exerting substantial impacts on athletic performance. Notwithstanding these multifaceted influences, TENG-based technologies demonstrate distinctive advantages in objectively quantifying human sport through real-time monitoring of joint motion potentials and other quantifiable electrophysiological metrics, independent of psychological indicators, thereby exhibiting demonstrated reliability and reproducibility. Furthermore, the inherent temporal latency associated with conventional psychological assessment methodologies (e.g., psychometric inventories or neuroimaging modalities) renders them incompatible with real-time monitoring requirements critical for applications such as competition officiation assistance. Moreover, given the current engineering-oriented exploratory phase in intelligent sports research, priority should be given to establishing biomechanical benchmark models, which will lay essential theoretical groundwork for future multidimensional integration of human motion determinants, including psychological factors. Consequently, this paper concentrates on the creation of self-powered intelligent sports equipment, examining its potential uses in physiological data monitoring, training support, event refereeing, and sports injury prevention, and rehabilitation through an intelligent sports perspective.

### Physiological Data Monitoring

Physiological indicators form the foundation for evaluating an athlete’s competitive readiness. These indicators encompass biochemical indicators such as heart rate, blood pressure, blood lactate level, and hemoglobin concentrations, among others. Additionally, they include physical fitness metrics like muscle strength, endurance, and flexibility. For instance, it is imperative that a long-distance runner maintains their heart rate and blood pressure within specified limits during training to ensure that the body can endure the exertion. Concurrently, stable hemoglobin levels are crucial to guarantee effective oxygen delivered to the muscles. The effective acquisition, recognition, and analysis of motion information are fundamental to intelligent movement. Such capabilities can aid athletes in honing their skills and in formulating scientifically backed training regimens and competition strategies. The volume of research studies on TENG-based physiological monitoring continues to grow due to its immense potential in next-generation sports/clinical technologies for daily activity, athletic performance, and health status monitoring.

**Fig. 3:**
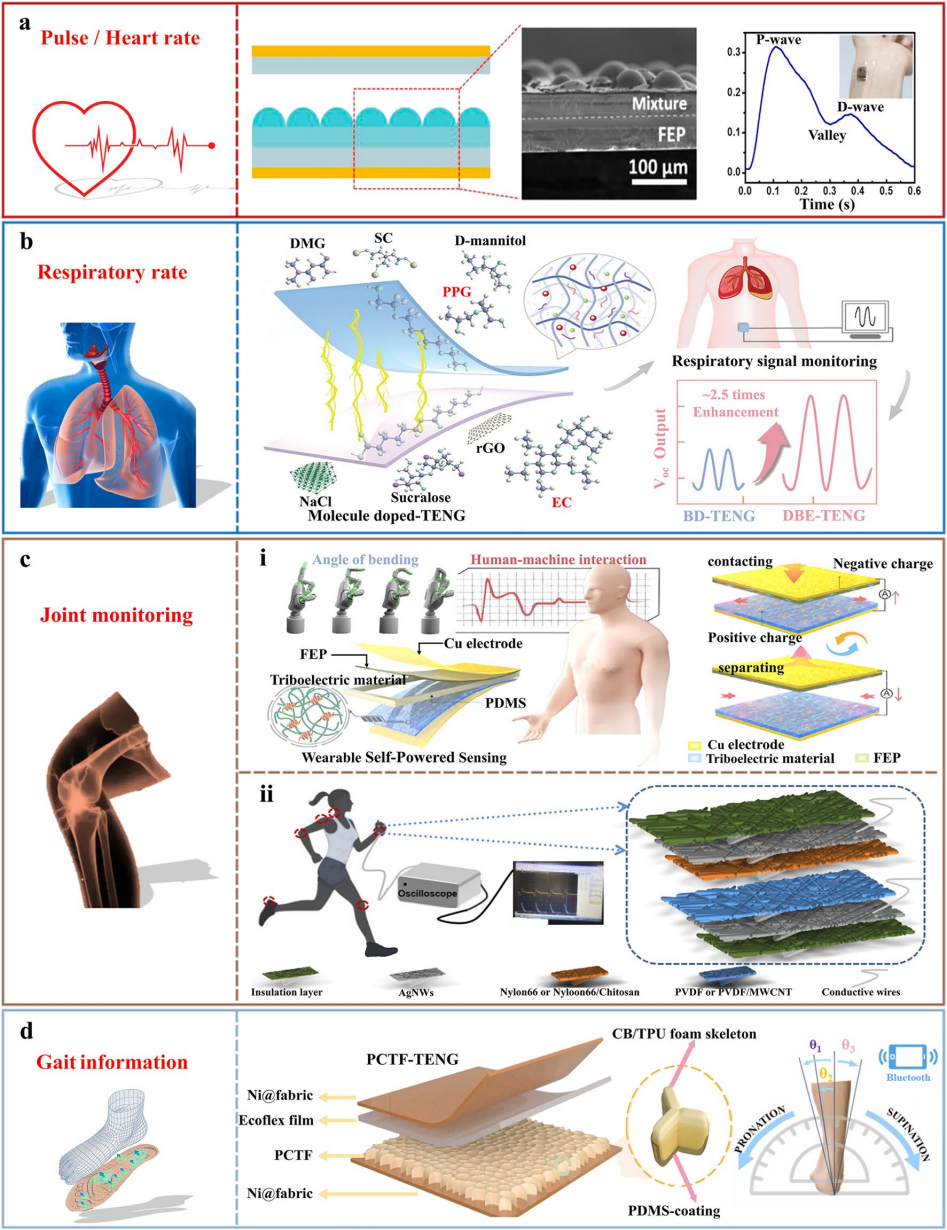
Application of TENG-based physiological data monitoring equipment. **a** Expandable microsphere-based TENG for respiratory and pulse monitoring. Reproduced with permission [37]. Copyright 2019, Elsevier. **b** Application of DBD-TENG to respiratory rate monitoring. Reproduced with permission [38]. Copyright 2024, Wiley–VCH GmbH. **c** TENG-based motion monitoring equipment for joint information: **c-i** schematic diagram of the composition and application of TENG based on polymer triboelectric material. Reproduced with permission [41]. Copyright 2024, American Chemical Society. **c-ii** SWS-TENG assembly diagram. Reproduced with permission [42]. Copyright 2024, Elsevier. **d** Schematic of the structure of the PCTF-TENG for gait monitoring. Reproduced with permission [43]. Copyright 2024, Elsevier

Heart rate and pulse serve as critical indicators of exercise intensity. By continuously monitoring these vital signs, athletes can ascertain whether their current workout intensity is moderate and whether they are achieving their desired training outcomes. For instance, during aerobic exercise, maintaining an optimal heart rate zone can significantly enhance cardiorespiratory fitness and endurance. Conversely, anaerobic exercises necessitate a higher heart rate to facilitate high-intensity muscle activity. Liu et al. [37] developed a self-powered pressure sensor by incorporating expandable microspheres into polydimethylsiloxane (PDMS), a cost-effective, easily processable, and 100% sensitive material for monitoring bio-signals. The microspheres/PDMS mixture was initially spin-coated onto a flat surface/substrate to serve as the triboelectric layer. Upon heat application, the microspheres expanded, leading to the emergence of microstructures on the otherwise flat PDMS surface, as shown in Fig. 3a. The sensitivity of the prepared sensor increases in correlation with the quantity of microsphere content, reaching the maximum value of 150 mV Pa^−1^ at a weight percentage of 1%. A comprehensive theoretical analysis on the output voltage has been provided, which shows a high level of alignment with the experimental results and provides guidance for device optimization. The pressure sensor is sensitive enough to respond to even subtle body deformations. Once affixed to a human subject, the wearable sensor proves its capability to operate autonomously as a self-powered monitor, capable of detecting subtle physiological signals such as respiration or pulse when affixed to the chest and wrist. Alongside heart rate, these capabilities can provide additional data regarding systolic, diastolic, and ventricular pressures.

Respiration serves as a critical metric in the realm of sports monitoring. The rate and depth of breathing can provide insights into the body’s oxygen consumption and lung function. By diligently monitoring these aspects of respiration, one can evaluate the effects of exercise on the respiratory system. This not only ensures the optimal functioning of the respiratory system during physical exercise activity but also aids in the prevention of exercise-induced respiratory complications. Respiratory monitoring sensors often come into prolonged contact with the human body, and may sometimes be fully or partially implanted. As such, materials that are not biocompatible or biodegradable can cause physical irritation or chemical reactions in human tissues, leading to rejection reactions, inflammation, and other adverse effects. However, TENG-based respiratory monitoring sensors, which are biocompatible and biodegradable, can avoid these potential risks due to their ability to be absorbed or metabolized by the human body after use. This not only enhances user comfort but also ensures the safe application of these devices. The challenge lies in effectively distinguishing respiratory frequency and obtaining relevant information, given the extremely low vibration energy generated during breathing. Thus, there is an urgent need for triboelectric materials that possess biocompatibility, biodegradability, and high-output performance for TENG-based respiratory monitoring sensors. Molecular doping is a facile but effective means to change the materials’ triboelectrification properties and improve the output performance of TENGs. However, there is a lack of regular and mechanistic studies on the differences in triboelectrification properties of different doped molecules, especially those with biodegradability and biocompatibility. Li et al. [38] selected eight biocompatible doped molecules as electropositive and electronegative materials in terms of biocompatibility, biodegradability, chemical group or elemental characteristics, and triboelectrification properties. The changes in the triboelectrification properties after doping and their varying trends were investigated in detail. Notably, after doping small amounts of low molecular weight polypropylene glycol (PPG) and ethyl cellulose (EC) into electropositive and electronegative biodegradable polymers (BPs), the open-circuit voltage (*V*oc), short-circuit current (*I*sc) and transfer charge (*Q*sc) of the dopped BD-TENG (DBD-TENG) were increased by 2.73, 2.60, and 2.44 times, respectively. The fabricated DBD-TENG has a multilayer structure: poly (propylene terephthalate carbonate) (PTMC) encapsulation layer, magnesium (Mg) electrodes, polyethylene oxide/polypropylene glycol (PEO/PPG) positive triboelectric layer, and polycaprolactone/cellulose (PCL/EC) negative triboelectric layer. The DBD-TENG has a high degree of flexibility and can be bent arbitrarily, and it shows a significant improvement in terms of the monitoring resolution of abnormal respiratory signals, as shown in Fig. 3b. This study not only provides theoretical guidance for the selection of triboelectric molecules but also provides a theoretical basis for an in-depth study of the triboelectric mechanism in the molecular level.

Joint monitoring provides athletes with insights into their joint stability, allows for an assessment of muscle strength and coordination, and enables the visualization of an athlete’s technique accuracy. It can also detect minor changes and abnormalities in an athlete’s joints in a timely manner, which can help mitigate potential injury risks. Figure 3c illustrates that in wearable triboelectric sensors, the meticulously calibrated interplay between strength and toughness in polymer materials is paramount. This is crucial to accommodate varying forces, ensure seamless compatibility with the user, and precisely detect alterations in the user’s motion [39, 40]. However, achieving both high strength and toughness concurrently remains a substantial challenge. Cai et al. [41] developed polymeric electrification materials with tunable mechanical properties by controlling the crystalline domains through ion-specific effects (Fig. 3c-i). The dense cross-linking of these domains restricts initial network deformation and boosts energy dissipation. This results in materials characterized by high tensile strength, toughness, an elongation at break of 416.7%, and a modulus of 545.0 MPa. Leveraging these properties, the contact area of the TENG gradually increases with an increasing bending angle, which leads to a rising trend in voltage output and enables the real-time feedback on human joint movement. The TENG fulfills the requirement for varying mechanical strengths or strain capacities in sensing materials for different human body positions. This ensures that both the human body and the flexible wearable sensor undergo identical strain, thereby guaranteeing precise signal acquisition. However, the relatively low current density output of TENG devices poses a limitation for their potential for miniaturization and use as portable power sources for implantable, wireless, wearable electronics, and appliances. Therefore, it is crucial to maximize the charge density output within a limited triboelectric layer area to enhance TENG performance. While much research has been directed toward enhancing the current density of triboelectric materials through surface modifications, there remains considerable room for exploration in terms of improving fiber triboelectric materials via dipole–dipole interactions. Enhancing current density in triboelectric materials through surface modification or material selection primarily targets the optimization of interfacial charge characteristics. By leveraging hydrogen bond-mediated dipole–dipole interactions, this approach strengthens interchain coupled polarization and promotes deep-layer electron migration within the material matrix. Such mechanisms enable efficient charge transfer from bulk material to contact interfaces, significantly improving current density. In an effort to substantially augment the TENG current density, Duo et al. [42] proposed an effective intermolecular bridging mechanism by using hydrogen bond mediated dipole–dipole interactions between chitosan and nylon66 molecular chains. Specifically, the dipole–dipole coupling formed via hydrogen bonds between chitosan and nylon66 molecular chains establishes intermolecular bridges, facilitating electron migration within the material and thereby enhancing the current density. Furthermore, the introduction of chitosan reinforces dipole polarization along the molecular chains, which optimizes the dielectric properties of the composite material and further improves charge separation efficiency. Figure 3c-ii shows the schematic representation of the self-powered wearable sensors based on TENGs, showcasing the structure of all the hybrid fiber membranes prepared through the electrostatic spinning process. This design ensured that the system was breathable and possessed antiviral characteristics. Additionally, varying proportions of chitosan were mixed with nylon66 to modify its affinity for positive charges. A minimal quantity of multiwalled carbon nanotubes was added into polyvinylidene fluoride (PVDF) to serve as an electron-absorbing counterpart, thereby preventing air breakdown. An insulating layer was also employed to inhibit the escape of electrostatic charges and to reduce the impact of moisture. The prepared TENG sensor can be affixed to the fingertips, wrists, and neck, making it convenient for monitoring human physiological signals during walking/running/standing.

Gait analysis provides invaluable insights for athletes and coaches, enabling them to comprehend the technical nuances of an athlete’s gait and pinpoint areas for improvement. In sprinting events, for instance, it aids in evaluating an athlete’s start reaction time, acceleration method, and sprint velocity, subsequently facilitating the refinement of technical movements during the start, acceleration, and sprint phases. For long-distance running, gait analysis offers enhancements in running efficiency by examining cadence, stride length, and running economy. Moreover, in jumping and throwing events, it assists athletes in optimizing their techniques during jumping, aerial movements, landing, and the transition and throwing phases. Given these applications, there is a pressing demand for highly compressible strain sensors that exhibit exceptional sensitivity, suitable for intricate external pressure conditions. The challenge of creating rational sensor structures that can achieve both low detectable limits and wide linear-sensing range persists. Ding et al. [43] developed a robust, anisotropic conductive PDMS/carbon black/thermoplastic polyurethane foam (PCTF) using directional solidification and ultrasonic impregnation methods. The unique orientation structure of the PCTF yields exceptional high triboelectric output and heightened sensitivity, making it advantageous for strain sensing. Furthermore, it can detect motion states and running postures. Figure 3d illustrates the construction of a PCTF-TENG with superior self-powered sensing performance, using an Ecoflex film (an electronegative material), Ni@fabric (an electrode) and PCTF (an electropositive material). The thermoplastic foam skeleton contributes directly to the self-powered sensing process. Encasing the foam skeleton with PDMS effectively inhibits the shedding of carbon black nanoparticles in contact-separation mode. The PCTF-TENG is capable of distinguishing between diverse locomotor activity of walking/running/jumping, indicating potential applications in rehabilitation training and strength restoration. The intelligent insole system, comprising a plantar array of PCTF-based strain sensors (I-PCTFS), is designed to enable the wireless sensing device to visualize the landing time of each step and dynamically monitor the athlete’s movement status.

At present, the majority of TENG-based intelligent sports monitoring devices are limited to tracking basic physiological signals such as pulse and heart rate. The precision, accuracy, and feasibility of monitoring more intricate physiological signals, like muscle activity and joint angle, require significant enhancement. For instance, lactate concentration in sweat is a pertinent biomarker during exercise. While carbon fiber fabric integrated with TENGs has been suggested as self-powered sensors for lactate detection due to its excellent selectivity and sensitivity, there remains an absence of analytically validated wearable systems substantiate this claim [44, 45]. Additionally, wearable devices often gather personal data and private information from users. TENG-based smart devices are no exception, necessitating stringent measures to ensure the secure storage and legal utilization of data in real-world applications.

While TENG-based sports monitoring devices offer the advantages of lightness and softness, practical implementation requires consideration of user acceptance and comfort. Prolonged wear may induce skin discomfort or pressure, thereby negatively impacting the user experience. Additionally, the device’s configuration—including shape, dimensions and materials used—must be tailored to satisfy individual user requirements. Potential interferences such as mechanical vibration and electromagnetic disruptions can degrade signal quality and affect the accuracy of monitoring results. Although TENG technology can be applied to a variety of textile structures such as plain cloth and twill cloth, enabling flexible physiological signal monitoring from different locations, enhancing performance necessitates a complex preparation process. In particular, core–shell yarn preparation and coating technology require meticulous handling and control, increasing production costs and process complexity. This limits its ability for mass production and broad application.

The potential health and safety concerns associated with TENG-based wearable devices primarily stem from prolonged skin contact and the potential for electromagnetic interference (EMI). To address these issues, we propose the following mitigation strategies:

1. *Biocompatible materials* The use of biocompatible and hypoallergenic materials in the fabrication of TENG-based devices can minimize skin irritation and allergic reactions during continuous wear.
2. *Low-power design* TENGs inherently operate at low power levels, which significantly reduces the risk of electromagnetic interference with other medical devices or physiological systems.
3. *Ergonomic design* Optimizing the devices’ structural design to ensure comfort and minimize mechanical stress on the skin can further enhance user safety during long-term monitoring.
4. *Rigorous testing* Prior to deployment, TENG-based devices should undergo rigorous biocompatibility and safety testing to ensure compliance with international standards (e.g., ISO 10993 for biological evaluation of medical devices).

### Sports Training Aids

Technical performance is a critical component for evaluating an athlete’s competitive standing, encompassing the accuracy, fluidity, and coordination of their movements throughout a competition. Additionally, it examines the athlete’s capability to adjust optimally in response to changing competition scenarios. For instance, in gymnastics, competitors might be tasked with executing a series of complex movement sequences during a competition. The quality of these movements directly influences their final score. The rapid development of information technology in recent years has led to a significant transformation in the landscape of sports monitoring, with the emergence of intelligent sports as a prominent development trend. The core of intelligent sports lies in the effective monitoring of motion and the ability to discern different movements or techniques. This allows for the quantification of an athletes’ motor behavior and movement patterns, thereby enhancing their skills and the formulation of evidence-based training plans and competition strategies [46, 47]. TENG has been successfully employed to construct multifunctional sensors capable of measuring force, velocity, acceleration, direction, and angle, demonstrating its considerable significance in the field of smart motion monitoring [48–51].

**Fig. 4:**
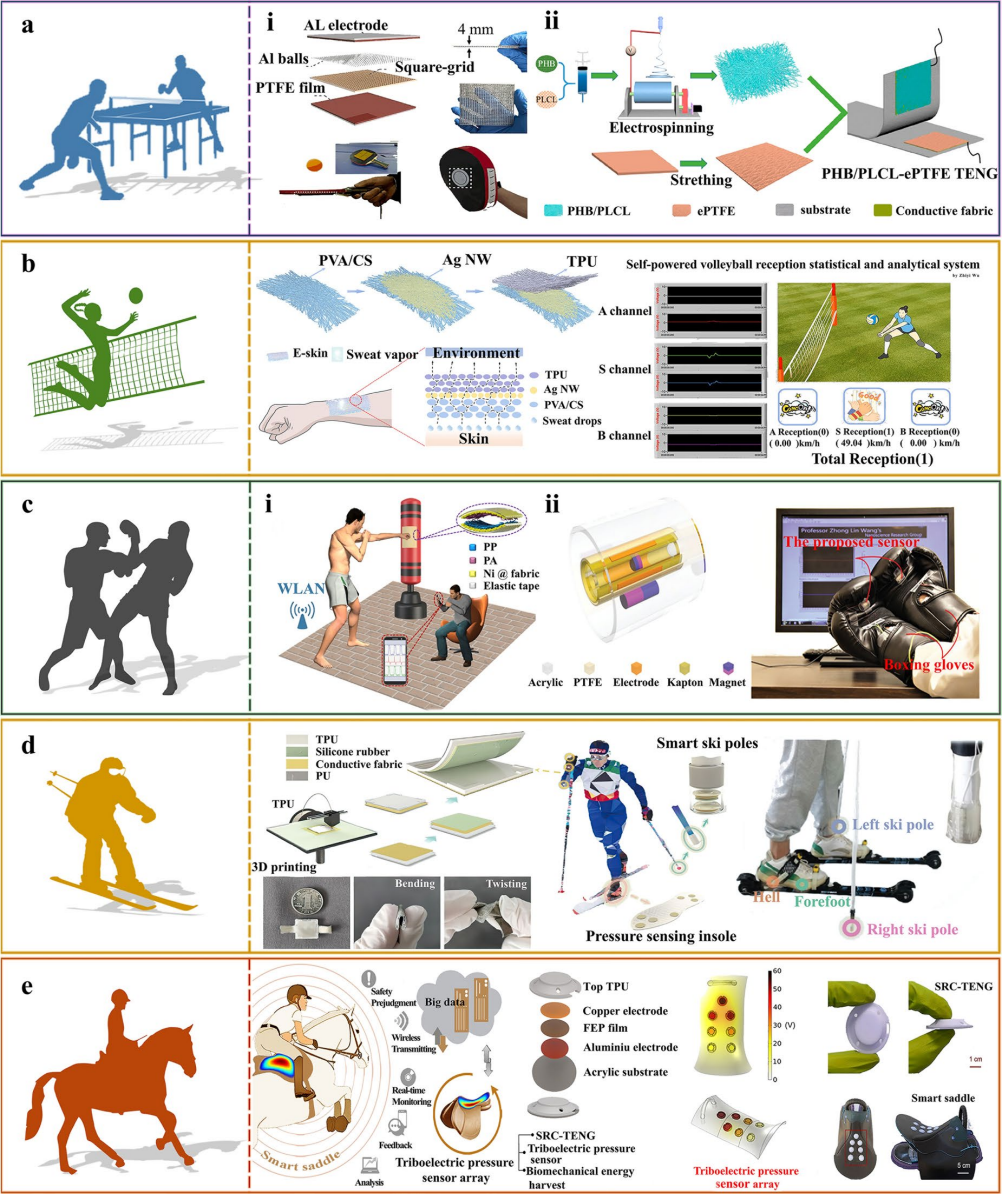
Application of TENG-based sports training aiding devices. **a** Application of TENG-based sports monitoring equipment in table tennis training assistance: **a-i** Schematic of SG-TENG structure and its integration into racket and boxing gloves. Reproduced with permission [53]. Copyright 2017, Tsinghua University Press and Springer-Verlag GmbH Germany. **a-ii** Process flow diagram for the preparation of PHB/PLCL-ePTFE TENG. Reproduced with permission [54]. Copyright 2023, American Chemical Society. **b** E-skins applied to self-powered sensors and volleyball serve-receive statistical analysis systems. Reproduced with permission [58]. Copyright 2021, American Chemical Society. **c** Application of TENG-based sports monitoring equipment in boxing training assistance: **c-i** SP-PTM for boxing training monitoring. Reproduced with permission [60]. Copyright 2019, Elsevier. **c-ii** Structure of MS and its application in boxing punch velocity monitoring. Reproduced with permission [61]. Copyright 2019, WILEY–VCH Verlag GmbH & Co. KGaA. **d** The structure and making of CF-TENG and its application to cross-country skiing monitoring. Reproduced with permission [62]. Copyright 2022, Science China Press and Springer-Verlag GmbH Germany, part of Springer Nature. **e** Structural design and schematic diagram of the SRC-TENG for application in equestrian sports. Reproduced with permission [65]. Copyright 2022, American Chemical Society

Due to the application environment of wearable sensors in motion monitoring, they should not affect human activities, so they must be flexible and lightweight. Specifically, currently used TENG-based flexible sensors mostly made of square flat films, which are usually attached to the human body (skin and clothing) or large sports facilities. Due to the different shapes and spatial structures of different sports facilities, sensors must meet the corresponding personalized requirements. However, conventional thin-film-based sensors are difficult to meet the requirements of personalized spatial structure and flexibility, and conformal attachment to the 3D surfaces of sports facilities, which limits their large-scale applications [52]. Accordingly, TENG sensors should have their own spatial structure and customization to meet various monitoring demands. Figure 4a-i depicts a 3D-printed square grid TENG (SG-TENG) structure with aluminum (Al) spheres designed by He et al. [53]. The square grid structure of the SG-TENG can harvest vibration energy across a broad frequency range at different vibration angles (0°, 45°, 90°) and work at different vibration angles. The unique design of the SG-TENG is conducive to its expansion and integration into other structures. When two SG-TENGs are connected in parallel, there is a significant increase in *V*_OC_ and *I*_SC_ across the entire vibration-frequency range. The integration of the SG-TENG into a focusing glove as an impact sensor has potential applications in various combat sports (e.g., boxing and kickboxing) to detect the frequency of punches and kicks, as well as track the force of each punch delivered practitioners. By analyzing the obtained data, athletes have an opportunity to review their performance and refine their skills during subsequent training sessions. Given its portability, lightweight nature, scalability, and ease of integration, the SG-TENG holds considerable promise for applications in energy harvesting and sensing. In an endeavor to create stretchable and highly sensitive eco-friendly monitoring devices for assessing individuals’ exercise health and postural norms, Wang et al. [54] pioneered the use of biocompatible poly(lactic-caprolactone) (PLCL) to enhance electrospun poly(hydroxybutyric acid) (PHB) membranes, and prepared PHB/PLCL composite membrane with varying PLCL concentrations, as illustrated in Fig. 4a-ii. The incorporation of PLCL endowed the electrospun composite membranes with superior tensile properties. Notably, the elastic recovery of the composite film at a 40% strain reached 68% of its stretch as the PLCL content increased. This composite film exhibited a remarkable toughness enhancement of 546%, showcasing exceptional super toughness and tensile characteristics. A highly stretchable large deformation sensor, constructed from an electrostatically spun film with a PLCL content of 50% was designed to precisely discern motion types and the nuances of the batting stance. The PHB/PLCL-ePTFE TENG can adeptly differentiate between the different techniques used in table tennis and badminton, such as stroke, backhand, and snap postures, offering a fresh approach to large deformation monitoring. Moreover, the proposed TENG sensor holds potential for the monitoring of other sports characterized by distinct contact and separation dynamics.

In order to perform real-time sensing, wearable devices must exhibit elasticity and flexibility for comfortable attachment on the skin during a broad range of sporting activities, wherein taking into account the inevitable distortions that occur therein. Notably, inadequate breathability and a propensity for bacterial growth can cause discomfort or even disease, which may present as itching and inflammation [55–57]. As body temperatures rise and sweat accumulation during exercise, these factors can adversely affect the effectiveness and progression of sports. Consequently, wearable devices intended for sports monitoring must demonstrate high levels of breathability, antimicrobial properties, and operational stability. Shi et al. [58] presented a self-powered statistical and analytical device for volleyball sport sensing, utilizing a flexible/breathable/antimicrobial E-skin based on TENG. The silver nanowire (AgNW) electrodes were positioned between the poly(vinyl alcohol)/chitosan (PVA/CS) substrate and thermoplastic polyurethane (TPU) sensing layer through alternating electrostatic spinning and spraying techniques. This process led to the creation of a porous structure characterized by a mesh network and three-dimensional laminates, as illustrated in Fig. 4b. Notably, the E-skin material demonstrated superior moisture absorption and breathability, with a rate of 10.32 kg m⁻^2^ day⁻^1^. This characteristic ensures optimal thermal and humidity comfort during wear, without compromising the stability and durability of the electrical output. Systematic evaluation under controlled conditions (40% relative humidity, 38 °C) at simulated speeds of 65, 95, and 125 km h⁻^1^ revealed stable (*V*_OC_) maintenance over 1200 continuous operational cycles, confirming its exceptional operational durability. Comparative analysis with state-of-the-art studies indicates that the E-skin achieves a high sensitivity of 0.3086 V kPa^−1^ within a sensing range of 6.65–19.21 kPa. Notably, while the *V*_OC_ exhibits a gradual decrease with increasing relative humidity (30%-80%), it remains stable across the physiological temperature range of 36.5–38 °C. These outstanding characteristics in electrical output stability and durability establish a reliable foundation for the application of wearable self-powered sensing systems in sports statistics and performance analysis. The effective execution of serve-receive plays an integral role in modern volleyball, especially with regarding to a team’s tactical deployment. Generally, the serve player’s role is to disrupt the opposing team’s overall tactics by serving to their attackers or altering the ball’s speed during play. The serve player also coordinates a strategic blocking defense and interferes with the opposing team’s reception. Therefore, teams have dedicated substantial effort to improve their serve-receive training and revise their technical and tactical systems. In this context, Shi et al. developed a 2 × 3 electronic skin array for serve-receive perception testing at different hitting positions and further created a statistical analysis system for serve-receive situations in volleyball matches, which includes motion-perception, position-monitoring, and distribution-statistics functionalities. The system enables athletes and coaches to recognize and rectify their team’s shortcomings, thereby aiding in the development of effective game strategies. This study suggests that integrating wearable electronics combined with self-powered sensors could enhance the practical utilization of this technology in the field of sports.

The performance of TENG sensors depends on several parameters, such as contact affinity, structural design and the potential difference between two triboelectric layers during contact-separation process [59], as shown in Fig. 4c. To improve the contact affinity between the triboelectric layers, Peng et al. [60] proposed a large-area, durable, and low-cost fabric-based TENG (FB-TENG) by using melt-blown technology, as illustrated in Fig. 4c-i. The FB-TENG consists of three main components: polypropylene non-woven fabric (PP-NWF), commercial polyamide 66 fabric (PA-66-F), and conductive fabric (Ni fabric). Moreover, the device shows outstanding stability over a long period, which is suitable for various applications. The FB-TENG can serve as a self-powered fast-responsive sensor for accurately monitoring boxing training. This study presents a sophisticated approach to simply, sustainably and inexpensively develop FB-TENG, which has promising applications in self-powered tactile sensing, motion tracking, and remote wireless training monitoring systems. The magnetically regulated TENG multifunctional sensors are capable of measuring variety of movement parameters, such as acceleration, velocity, and direction, applicable to both rotary and linear motion. Wu et al. [61] developed a self-powered cylindrical multifunctional sensor (MS) that converts the kinematic state of a magnetic column into electrical signals. This MS is capable of detecting acceleration, force, and rotation parameters. As illustrated in Fig. 4c-ii, the MS consists of a translational-rotational magnetic component, a TENG module, and an acrylic housing, which can convert translational motion into either an oscillating motion of a low-damping magnetic column around a triboelectric layer or a multiturn rotational motion to drive the TENG to produce a voltage output. In order to enhance the output of TENG, it is better to choose electrode materials with low resistance, small work function, and an appropriate surface morphology. The magneto-strictive rotary mechanism, owing to its structural characteristics, is highly responsive to subtle impacts and facilitates the monitoring of rotational motions without necessitating coaxial mounting. Furthermore, an application program has been developed to measure the acceleration of punches thrown by a boxer, the hitting force of a golf club, and the swing angle. This serves to validate the feasibility and effectiveness of the method, showcasing its immense potential in the realm of intelligent sports.

Correcting the structural deficiencies by innovative fabrication methods and a systematic design approach to make an optimally compatible TENG is a crucial step toward portable, adaptable, and real-time self-powered sensing systems for the human body. Yang et al. [62] presented a customizable and flexible TENG (CF-TENG) with 3D-printing thermoplastic polyurethane (TPU, in the form of an elastic shell) as the triboelectric layer, which exhibited a robust correlation with external pressure due to the unique spatial architecture (Fig. 4d). The CF-TENG weighs only 0.34 g, light enough to hold in one’s hand. In practical applications, it exhibits remarkable sensitivity to movements of the finger, wrist, and elbow joints. When the external force increases from 2 to 55 N, the output voltage gradually rises from 2.9 to 12.8 V, and the output current increases from 28.4 to 567.5 nA. The output voltage is stable, reliable, and proportional to the external voltage, and shows repeatable performance in response to bending of the joints. When the CF-TENG is used in insoles or ski poles, it needs to be waterproofed. The utilized TPU has high elasticity, water repellency, antimicrobial properties, and is non-toxicity to human skin, which ensures the relevant requirements. Experimental results indicate that regular variations in temperature and humidity in daily environments have negligible effects on the output performance of the CF-TENG. After 2500 cycles of testing, the output voltage and current remain stable. Thus, even under varying working conditions, using voltage as a force-sensing parameter proves to be effective and reliable. Thus, an advanced motion monitoring system was developed by combining pressure-sensing insoles equipped with seven sensor units, and ski pole sensors. This system captured detailed plantar pressure distribution and ski motion data, such as the frequency and force of ski pole impacts. The gathered signals undergo feature extraction using both the subspace K-nearest neighbors (KNN) and the novel P-Find’s integration algorithm. As a result, the system can distinguish human movements of walking/tiptoeing/running/jumping with a remarkable 98.2% accuracy. Moreover, for cross-country skiing, insights into the skier’s techniques, including the specific subtechniques used across different track segments, the frequency and force of ski pole strikes, and the total number of steps, are invaluable for both coaches and skiers [63, 64]. The system demonstrated 100% accuracy in classifying the three subtechniques of classic cross-country skiing (diagonal stride, double push-up and knee-hold). Such a sophisticated level of accuracy can significantly aid coaches and athletes in scrutinizing various facets of training loads, and movements thereby facilitating the empirical modification of strategies to augment skiing proficiency. This research presents a novel approach to the personalized TENG application within the realm of intelligent sports, maintaining the integrity of the original content while enhancing its academic tone and readability.

For more sophisticated kinematic analysis, Hao et al. [65] outlined a simple yet efficacious method for fabricating self-rebounding curved surface TENGs (SRC-TENGs), which exhibit outstanding mechanical properties (Fig. 4e). The flat top of the design ensures complete and direct contact between two triboelectric layers, while the curved sides enable swift recovery from deformation across a wide pressure range upon release. The design is distinguished by its simple form, ease of fabrication, and compact size. Additionally, it demonstrates strong mechanical properties that contribute to the device’s reliability and durability in outdoor environments. The device’s efficient and unambiguous design enables common materials to be converted into a highly durable micromechanical energy harvester, with over 3000 cycles and superior mechanical properties, such as enhanced elasticity and stability. Integrating the SRC-TENG array on a saddle has enabled the development of a self-powered system capable of sensing riding characteristics. This system can provide real-time data and safety predictions to riders and coaches with a response time of ~ 16 ms. The SRC-TENG has demonstrated a resolution comparable to that of traditional motion sensors, while offering the benefits of being lightweight, easy to install, and energy-efficient for safety prediction systems. The successful application of SRC-TENG in smart saddles could potentially catalyze the advancement of TENG in various fields, such as micro-biomechanics, energy harvesting, and sensing. Furthermore, it presents the opportunity for self-powered systems to be utilized in competitive sports, intelligent sports facilities, and sports safety. This could further broad the application of self-powered systems in intelligent sports monitoring and assistance. This innovation represents a significant leap in the development of smart equipment for self-powered sports monitoring, setting the stage for distributed, portable, and real-time analysis.

The effectiveness of TENG-based intelligent sports monitoring equipment is evident in the realm of athletes’ daily training and competition analysis. By collecting and analyzing data from athletes during training sessions and events, coaches and athletes can better understand an athlete’s physiological state, technical capabilities, and potential limitations. This facilitates the formulation of more evidence-based training regimens and competitive strategies. With technological advancements, integration of TENG wearable devices is on the rise, suggesting a tighter amalgamation of its components, such as sensors, processors, and communication modules, to yield a more streamlined and efficient system. Another prominent trend in the development of TENG wearable smart devices is the miniaturization of the key components. The utilization of advanced fabrication techniques and materials enables the reduction in the device size and weight, which in turn minimizes interference with an athlete’s movements and enhances the comfort of wear. Nevertheless, conventional fabrication methods and material choices are inadequate to meet individual customization requirements. Furthermore, the challenge remains to produce large-scale TENG devices in a cost-effective, simple, continuous, and environmentally friendly manner. TENG-based sports equipment necessitates innovative strategies to enhance performance and optimize structural design. The research and development (R&D) and production costs can be substantial, potentially hindering its widespread commercialization and popularity. Nevertheless, there is significant potential for further advancements in this field. It is important to highlight that the capabilities of TENG wearable devices are in a state of constant expansion. Beyond foundational functions such as energy harvesting and physiological signal monitoring, future iterations may incorporate real-time data analysis, personalized training recommendations, and exercise status feedback. Although TENG technology introduces an innovative paradigm for wearable devices, it necessitates a phase of market acceptance. Both athletes and coaches might approach the new technology with a measured caution regarding its performance and efficacy, which could pose challenges in terms of marketing TENG devices.

Although TENG can harvest energy from human motions, its low energy conversion efficiency may limit its use in high energy consumption scenarios such as sports training assistance functions. This limitation confines its application in complex, precise motion data analysis and real-time feedback systems. Furthermore, TENG data collection might be prone to errors, particularly during extended, strenuous athletic training or in extreme conditions. In these situations, TENG devices may encounter stability and durability issues due to environmental factors like sweat and friction, potentially compromising the accuracy and reliability of the data derived from sports training. This could, in turn, affect the training program’s effectiveness and athlete performance. The future of TENG wearable devices is likely to feature the use of more pliable and permeable materials, alongside more intuitive designs. This ensures that athletes do not experience discomfort during extended periods of wear. Additionally, the interactive interface of such devices will become more user-friendly and intuitive. It is essential for TENG-based smart devices to incorporate more user-friendly operation methods, such as voice control and gesture recognition, to enable more convenient operation for athletes.

In addition to these improvements, we have also emphasized the importance of developing standardized testing protocols to evaluate the long-term stability and durability of TENG-based devices under realistic sports conditions. Such protocols should include accelerated aging tests, mechanical fatigue tests, and environmental stress tests to simulate the effects of prolonged use in harsh conditions. By addressing these challenges through a combination of material innovation, design optimization, and rigorous testing, TENG-based devices can achieve the reliability and robustness required for real-world sports applications.

### Assisting Refereeing in Sport Event

The rapid development of the IoT has underscored the importance of digitizing athlete data, particularly in the context of sports competitions. This trend has facilitated the integration of progressive technologies aimed at optimizing the performance of referees and ensuring the seamless execution of sporting events. The most objective criteria for ascertaining an athlete’s competitive standing are their performance results, encompassing factors such as ranking, scores, and comparative data with adversaries. For instance, in track and field events, an athlete’s performance data, metrics like running time and long jump distance directly influence an athlete’s ranking and competitive assessment. Despite pressure sensors, image sensors, and other such devices have become commonplace in these settings, challenges persist in terms of their durability and energy sources. Currently, the majority of sensors employed in sports are passive, including capacitive [66, 67], resistive [68, 69], and piezoresistive [70, 71] types, all of which require an external power source to function. Furthermore, the predominant Hawk-Eye system consists of high-speed cameras that entail significant acquisition and maintenance costs, and are susceptible to lighting and analog errors. Consequently, the TENG-based intelligent referee assistance system addresses these drawbacks, offering innovative opportunities for intelligent officiation in sports events.

**Fig. 5:**
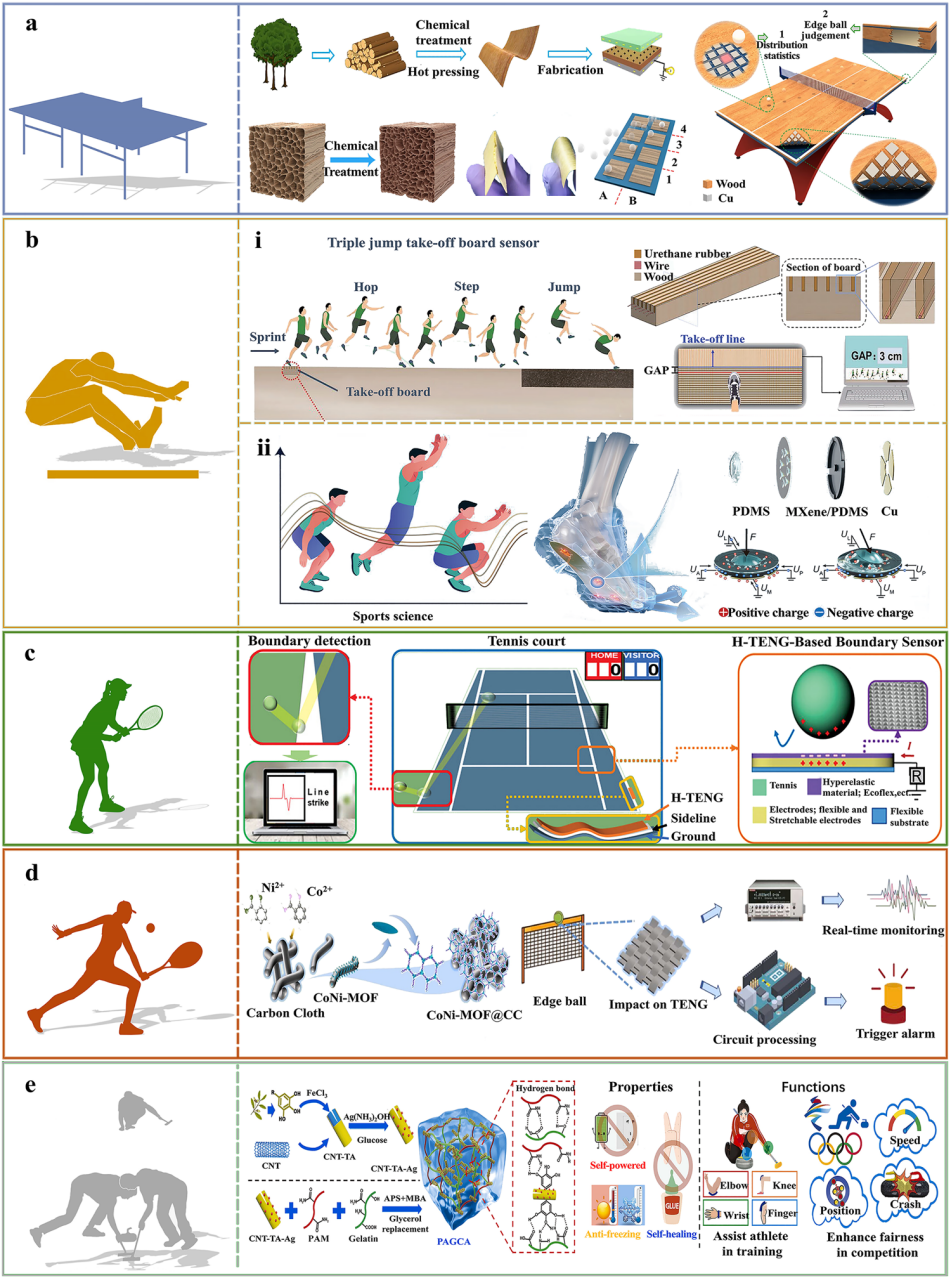
Application of TENG-based refereeing equipment for sporting events. **a** Schematic of the flexible wooden TENG and smart table tennis table. Reproduced with permission [77]. Copyright 2019, The Author(s). **b** Application of TENG-based sports monitoring equipment in long jump events. **b-i** Diagram of TBS integrated application and structure. Reproduced with permission [78]. Copyright 2022, Tsinghua University Press. **b-ii** Schematic diagram of TENG-based self-powered sensing insole. Reproduced with permission [79]. Copyright 2024, Wiley–VCH GmbH. **c** Working schematic of the boundary sensor system based on hyperplastic TENG (H-TENG). Reproduced with permission [82]. Copyright 2023, Wiley–VCH GmbH. **d** Schematic diagram of the composition strategy of CoNi-MOF@CC and the self-powered tennis match monitoring system based on C-TENG. Reproduced with permission [83]. Copyright 2024, Elsevier. **e** Schematic diagram of PAGCA synthesis and the performance and functions of the intelligent violation detection system. Reproduced with permission [84]. Copyright 2024, Elsevier

In the context of modern social development, it is imperative to simultaneously pursue intelligence and green sustainability. Currently, the majority of the materials utilized in TENG are non-biodegradable synthetic polymers, potentially leading to considerable environmental contamination [72]. Wood, one of the most abundant resources on Earth, is a sustainable/renewable/biodegradable material extensively employed in various fields such as construction, water purification, soft electronics, and energy harvesting/storage [73–76]. However, the mechanical and triboelectric properties of natural wood are not readily applicable in the fabrication of TENG. The only approach to modifying the properties of natural wood for the purpose of creating high-output TENG is through the application of effective processing techniques. Luo et al. [77] suggested a simple and effective two-step strategy for the preparation of wood films with excellent mechanical and triboelectric properties (Fig. 5a). Durable, high-performance, and flexible wood-based TENG (W-TENG) has been produced. The W-TENG is 7.5 times stronger than natural wood, more flexible, abrasion-resistant, and workable, and has an electrical output property that is over 70% higher than that of natural wood. In recent years, control of the ball landing point has been a key factor in determining the outcome of a game in modern table tennis. The flexible and durable single-electrode model W-TENG has been successfully employed in the manufacturing of intelligent table tennis tables, owing to its superior electrical performance. Additionally, a self-powered edge ball refereeing system was developed, proficient in speed sensing, motion trajectory tracking, and a statistical analysis of drop distributions. This system aims to offer both training assistance and real-time match guidance to players and referees. A comprehensive dataset comprising 10,000 impacts of the table tennis ball was amassed, with the statistical probability of each pixel meticulously recorded. Through a rigorous analysis of these statistics, insights into athletes’ movement habits can be discerned, subsequently informing their training regimens and facilitating the development of more effective game strategies. The wood material employed in the construction of the W-TENG retains the fundamental characteristics of a tabletop, a critical aspect for sports equipment. This research not only broadens the application domains of self-powered systems for intelligent sports monitoring and assistance but also paves the way for the potential integration of machine learning analytics in the intelligent sports industry. The statistical system for the distribution of table tennis landing points, based on W-TENG, showcases the possibility of creating innovative sports equipment for training purposes.

The successful integration of TENG with sports equipment provides a cost-effective method for real-time data monitoring, having potential applications in the field of smart sports (Fig. 5b). Xu et al. [78] developed an intelligent triple jump-starting board by using single-electrode mode TENG. The board consists of a slotted wooden board, wires to transmit the induced charge, and polyurethane rubber as a triboelectric layer (Fig. 5b-i). This intelligent self-powered springboard sensor (TBS), based on a solid wood substrate with a TENG, is utilized for accurate detection of triple jumpers’ jumping status, fulfilling the requirement for triple jump training judgment with up to 1 mm accuracy. A foul alarm system and measurement system were developed to monitor an athlete’s foot position relative to the take-off line, providing crucial data for both athletes and referees. Electrical signals generated by the TBS can be used to determine if an athlete has breached any rules. Moreover, the TBS’s ingenious design contributes to the evolution of new methods for fabricating TENGs. The unique configuration of the TBS offers promising avenues for devising novel TENG fabrication methodologies. Hu et al. [79] developed a self-powered sensing insole capable of comprehensively mapping the normal/shear plantar stresses using a TENG sensing array. As shown in Fig. 5b-ii, this array was meticulously crafted to align with the intricate anatomical structure of the human foot (first/fifth metatarsal head, midfoot, and heel). To decouple the asymmetric outputs of normal and shear stresses, four distinct TENG cells were strategically placed at the anterior/posterior/medial/lateral positions, respectively. The insole displays satisfactory static characteristics pertinent to plantar stress measurements. Furthermore, it adeptly monitored real-time fluctuations in plantar stress, effectively distinguishing varying states during activities such as the standing long jump. Such capabilities hold immense promise for enhancing sports performance analysis and rectifying underlying issues. This study pioneers the use of a self-powered insole that comprehensively senses both normal and shear plantar stress, marking a significant advancement with vast implications for sports science and medical research applications.

Despite the recent surge in popularity of TENG-based intelligent sports equipment, their relatively low resilience and limited environmental adaptability inhibit their detection sensitivity, rendering them unsuitable for use in complex settings [80, 81]. Consequently, there is an urgent need to develop self-powered sensors with high sensitivity, resilience, and durability for use in referee assistance applications in smart sports. Liu et al. [82] achieved complete super-elasticity of H-TENG by doping carbon nanotubes (CNTs) and carbon black with superplastic materials and incorporating a super elastic triboelectric layer. The principles of triboelectrification and electrostatic induction were employed to achieve boundary detection via the electrical output signal of the H-TENG, as illustrated in Fig. 5c. A comprehensive experimental validation and simulation analysis were conducted to investigate the stress–strain conditions of the H-TENG during high-speed collisions of tennis balls and its durability in terms of output performance. A system of auxiliary apparatus for the H-TENG has been developed and a series of practical demonstrations of the H-TENG’s applications have been conducted based on this system. When an impact occurs with the boundary, a triboelectric signal is generated in accordance with the TENG principles. This signal is subsequently amplified through a dedicated signal amplification module and captured by an integrated signal acquisition system and displayed on a computer. The referee can make a decision based on the field conditions and the details displayed on the computer screen, thereby enhancing the accuracy of the detection process. This research introduces an innovative and intelligent boundary detection in sporting events. It holds potential for wide-ranging application in addressing various challenges in sporting competitions, as well as in developing training methodologies for athletes.

Metal–organic frameworks (MOFs) are characterized by their high porosity, making them a promising choice for the cost-effective and efficient fabrication of TENGs. Their exceptional surface area, significant porosity, tunable pore size, and diverse functionality make them ideal for this application. As illustrated in Fig. 5d, Xiang et al. [83] successfully synthesized Co^2^⁺ and Ni^2^⁺-doped MOF-74 active materials (CoNi-MOF@CC) on carbon cloth using the solvothermal method. The resulting TENG (C-TENG) is both lightweight and wear-resistant, boasting a high V_OC_ and current density of 108.6 V and 4.11 mA m⁻^2^, respectively. The exemplary performance of C-TENG led to its adoption as a triboelectric sensor (C-TES) in sporting events, where it was integrated into a self-powering fencing performance counting system and a rubbing referee system. This self-powered sensing system based on C-TENG addresses the limitations of the current Hawk-Eye technology, which often results in extended intervals, thereby minimizing any adverse effects on athletes’ performances. The use of self-powered scoring reduces misjudgments, enhancing the fairness of the game. Moreover, this technology is also utilized to guide training and support real-time assisted matches, providing a new avenue to improve sportsmanship and further the integration of TENG in the intelligent sports industry.

In the context of the extreme conditions associated with winter events, there is a pressing need for sensors that can provide reliable data in the form of electric signals independent of external power sources. Besides, the sensors need to endure long-term low temperatures while functioning under harsh conditions. Figure 5e shows a self-healing, antifreeze, and antimicrobial nanocomposite hydrogel (PAGCA) developed by Tian et al. [84] using in situ acrylamide polymerization with Ag nanoparticles and gelatin, and tannic acid (TA) modified CNTs (CNT@TA@Ag) as a carrier. Experimental results demonstrate that after a 2-min self-healing process at 60 °C, the sensing performance of the PAGCA-based strain sensor remains comparable to its original state, indicating excellent self-healing capabilities. The prepared hydrogel strain sensor has a strain coefficient of 1.87 and self-healing efficiency of over 92%, making it suitable for accurate monitoring of an athlete’s training posture. PAGCA was embedded in PDMS to create a single-electrode TENG sensor to judge fouls, detect the position and speed of a curling stone, and identify collisions during curling matches. The PACCA-based TENG operates effectively at temperatures as low as −30 °C, allowing for the identification of rule infringements, the pinpointing of curling stone and speed, and the differentiation of collisions. Furthermore, the output voltage of the PAGCA-based TENG remains highly stable over 2000 contact-separation cycles, demonstrating exceptional long-term operational stability. The response and recovery times of the PAGCA-based TENG are recorded as 128 and 161 ms, respectively, highlighting its rapid response characteristics. To bolster the efficiency of athlete training and guarantee fair refereeing decisions in curling, a cutting-edge integrated system that merges wearable piezoresistive sensors with TENG sensors has been devised to detect violations. This study introduces an intelligent approach to identifying infractions during curling matches and showcases its potential uses in the prompt and unbiased enforcement of penalties, as well as in digital training for athletes across other sports.

Traditional human referees, when operating within the high-pressure, fast-paced environment of competitive sports, are often subject to misjudgments or missed calls as a result of subjective factors. The integration of TENG technology into both the infrastructure of sports facilities and athletes’ equipment can potentially mitigate these issues. This technology allows for real-time tracking of key parameters such as athlete movement, positioning, and speed, thereby enabling more effective, targeted coaching and training strategies. In ball games such as football and basketball, smart devices powered by TENG technology can support referees in making decisions on critical plays, such as determining whether the ball has left the field of play or touched a designated area, or if an offensive foul has been committed. By collecting and analyzing match data, these devices can provide referees with comprehensive and accurate information, fostering more equitable decision-making. Consequently, the use of TENG-based smart devices can reduce the influence of human error and enhance the fairness and precision of the competitions through objective data collection and analysis.

TENG technology has shown promise for sports event refereeing. However, challenges related to technical maturity and reliability remain. Current TENG-based smart devices might suffer from unstable performance and inaccurate data, requiring ongoing technical improvements. Moreover, refereeing aids have specific requirements depending on the sport, making them often bespoke to fit the particularities of the event. As a result, the development and production costs for these devices are expected to be high, potentially limiting their broad adoption in sporting events. Furthermore, using TENG-based smart devices involves collecting and analyzing significant amounts of athletes’ personal information and game data, raising privacy and rights concerns. Strong data protection measures and privacy policies are imperative to ensure data security and compliance.

### Sports-Related Injury Prevention and Rehabilitation

The correlation between an athlete’s workload, injuries, and subsequent performance necessitates the critical monitoring of athlete’s physical state within a high-performance sports environment. For instance, the muscle’s function extends beyond strength generation, it could be considered a crucial organ. The repeated stimulation from exercise induces changes in cellular structure and function, encompassing alterations in inflammatory and metabolic processes. Athletes aiming to maximize ‘fitness’ and minimize ‘fatigue’ require suitable monitoring tools to measure these outcomes [85]. Wearable sensors demonstrate considerable potential for application in sports injury prevention and rehabilitation, as they allow athletes and coaches to scrutinize individual body mechanics and modify techniques to mitigate injury risk or lessen exerted effort [86–88]. TENG-based intelligent sports monitoring equipment is broadly recognized and employed by researchers specializing in the field of sports rehabilitation and injury prevention, owing to the self-powered and portable attributes of this technology.

**Fig. 6:**
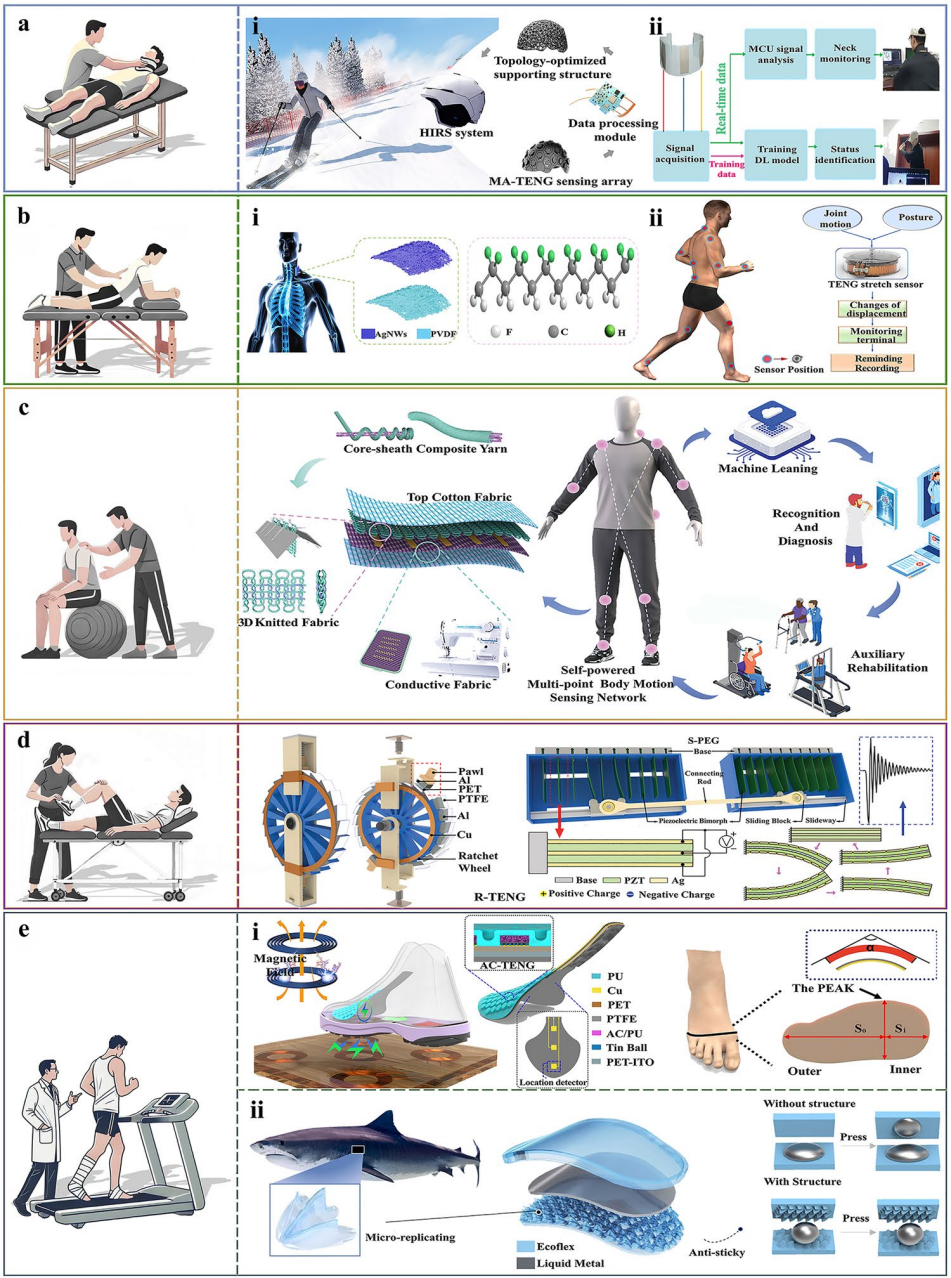
Application of TENG-based equipment for sports injury prevention and rehabilitation. **a-i** An MA-TENG-based head impact recognition system for real-time monitoring of head impacts. Reproduced with permission [91]. Copyright 2023, The American Association for the Advancement of Science. **a-ii** Workflow diagram of the NB-TENG-based smart neck monitoring system. Reproduced with permission [94]. Copyright 2023, Wiley–VCH GmbH. **b-i** The S-TENG is attached to the carotid artery for the monitoring of irregular heart rhythm. Reproduced with permission [96]. Copyright 2024, Elsevier. **b-ii** Stretch sensors for monitoring joint and spinal movements. Reproduced with permission [99]. Copyright 2021, The Author(s). **c** Schematic design of a textile-based self-powered multipoint human motion-sensing network (SMN) structure and a highly integrated gait recognition system. Reproduced with permission [101]. Copyright 2023, Wiley‐VCH GmbH. **d** Diagram of the In-Shoe Sensor Pad (ISSP) used in sports rehabilitation. Reproduced with permission [106]. Copyright 2021, Wiley–VCH GmbH. **e-i** Schematic diagram of the MC-EH-HL Lower Extremity Rehabilitation Sensing System. Reproduced with permission [109]. Copyright 2022, American Chemical Society. **e-ii** Bionic shark skin-based TENG gait abnormality sensor schematic. Reproduced with permission [110]. Copyright 2022, Elsevier

It is estimated that approximately 42 million individuals worldwide suffer mild traumatic brain injury (TBI) annually, especially in activities like skiing, American football, and boxing [89]. These mild concussive injuries, caused by sudden head impacts, are a significant, yet often overlooked, risk factor for various neurological disorders, including Alzheimer’s disease and other forms of neurodegeneration [90]. Multiwall carbon nanotubes as the nanofillers of polyurethane elastomers was proposed by Zu et al. [91] for avoiding the interfacial crack and the poor bonding between metals and polymers. A gentle curved sensation array composed of fully 3D-printing multiangle TENGs (MA-TENGs) were demonstrated for head impact monitoring, sports assistance and mild concussion prevention, as illustrated in Fig. 6a-i. MA-TENG is a metamaterial structure which can convert forces originating from all directions (compression/rotation/shear) into electrical signals without necessitating the presence of a power source. Experimental results demonstrate that the MA-TENG exhibits an average sensitivity of 0.214 V kPa⁻^1^, a response time of 30 ms, and a minimum resolution of 1.415 kPa, highlighting its excellent sensing performance. The MA-TENG shows stable sensing capabilities over a wide pressure range of 0–200 kPa. Furthermore, after 30,000 operational cycles, the overall sensitivity decreased by only 4%, further confirming the MA-TENG’s exceptional long-term stability and reliability. A head impact remote sensing system was built with objective assessment criteria. The MA-TENG sensing array and the corresponding deep convolutional neural network (DCNN) algorithms can be used for head impact monitoring in various sports of ice-skating/skiing/boxing/baseball/motorcycling with an accuracy of 98% for injury level assessment. The soft topology reduces the impact energy delivered to the brain thus providing certain protection to the athlete’s head from the impacts suffered during sports. The array employs TENG technology to convert kinetic energy from all directions into electrical signals, which are processed by an early warning system to reconstruct the head impact mapping and assess the injury severity. In addition, collection of standardized data will help create a data platform for further research on head impacts and mild concussions.

In high-impact sports, athletes often experience significant loads on the neck, necessitating its role in controlling head movement. The primary function of the neck muscles is to maintain stability in head position, thereby influencing an athlete’s performance and injury risk [92]. Cervical spine injuries represent one of the most severe types of damage across all sports, posing a significant risk to athletes. Consequently, the monitoring and prevention of such injuries is a valuable focus within sports medicine [93]. Sun et al. [94] have designed naturally biodegradable TENGs (NB-TENGs) using recyclable, sustainable, and inexpensive maize bracts for neck motion recognition (as shown in Fig. 6a-ii). The NB-TENGs employ a simple preparation process that is sustainable, cost-effective, and biodegradability without toxic by-products. System stability and output variation of the system are both below 2.3%, as determined from 4800 continuous simulations of the sensor at 4 Hz. A neck condition monitoring triboelectric sensor (NCM-TS) was created by integrating three NB-TENG sensors with elastic stretchable textiles to monitor neck stability during the golf swing. In specific motion states, the three channels produce voltage signals of particular amplitude and direction, facilitating the identification of neck motion. The NCM-TS was combined with a deep learning model to develop an intelligent behavioral monitoring system that can distinguish four distinct neck movements with ~ 94% accuracy. These sensors offer promising applications in detecting and correcting poor cervical spine posture, as well as in rehabilitation/healthcare training for neck disorders.

Breathing is considered a vital natural function integral to sustaining life [95]. It is the fundamental mechanism that enables the exchange of oxygen and carbon dioxide, essential for life. Proper breathing techniques can aid athletes in adjusting to the rigors of exercise, thereby improving performance, lessening fatigue, and preventing injury and muscle fatigue due to inadequate oxygen supply. Pan et al. [96] employed the electrostatic spinning technique to produce transparent, flexible, highly sensitive and high-power electronic skin based on TENGs (S-TENGs) (Fig. 6b-i). During heat treatment, the PVDF molecules rearrange to deflect dipoles and increase crystallinity, which produces more β-crystalline phases. These changes enhance the transparency of the PVDF films and the output power of the S-TENG. The resulting S-TENG-based E-skin has a high transparency of 80% and can detect light touches of 0.13 g with high sensitivity and operational stability. The device has a multilayered nanostructure with a complex network of 3D micron-to-nano graded pore network, allowing for substantial air permeability. The E-skin generates an open-circuit voltage of 301 V and a short-circuit current of 2.7 μA over an interaction area of 4 × 4 cm^2^ under an external force of 8 N, corresponding to power densities of up to 306 mW m⁻^2^. The S-TENG also pairs with signal processing circuitry, enabling continuous monitoring of respiratory signals and the differentiation of respiratory states at different exercise intensities when attached to a human neck. The S-TENG could provide tailored recommendations for future personalized health recovery and professional sports training.

The proper functioning of the spinal column requires a stable configuration. This stability is vital not only for protecting neural tissues from injury during routine physical activity, but also for allowing force transfer between the upper and lower extremities. Furthermore, it is essential for generating active force within the trunk [97, 98]. Li et al. [99] developed a small, high-precision tensile sensor for the real-time monitoring of human spinal motion, which can be worn by the user. This was achieved by combining a grating-structured TENG with a retractable badge reel, as illustrated in Fig. 6b-ii. The entire setup of three fabricated devices (diameter ~ 33 mm, a thickness ~ 10 mm, and a weight ~ 9.6 g) is encapsulated in a 3D-printed box. The small size and light weight of the box make it easy to attach to the skin or mount on vests. The device shows synchronous deformation with body’s movement, indicating high sensitivity (8 V mm^−1^), excellent sensing resolution (~ 0.6 mm), remarkable robustness (over 120,000 stretching cycles), and minimal hysteresis. Studies of common human activities and movements demonstrated the sensor’s ability to record various motions in real time, such as knee/arm bending and neck/lumbar torsion. The feasibility and accuracy of the sensor for monitoring spinal motion were confirmed by comparing its performance with commercial inclinometers and depth cameras. This lightweight, precise, and resilient tensile sensor could help reduce the risk of diseases caused by long-term and abnormal postural habits. It could also be used as a rehabilitation tool to monitor a patient’s joint movements in real time over a long period, aiding in their recovery from injury.

Elbow pain frequently affects athletes, especially those involved in throwing activities. Such injuries can occur due to acute trauma from a direct fall or chronic microtrauma. During throwing motions, excessive valgus extension can lead to chronic injuries with significant implications for both recreational and professional athletes [100]. Wei et al. [101] proposed a self-powered multipoint somatic motion-sensing network (SMN) constructed from a fabric monolithic structure for use in biometric recognition, gait analysis, and assisted rehabilitation training (Fig. 6c). The SMN is realized using traditional knitting methods combined with innovative digital embroidery techniques. Integrating TENG technology with traditional textile production ensures that the SMN possesses high-pressure sensitivity of 1.5 V kPa^−1^. It also boasts other notable properties such as complete flexibility, excellent air and moisture permeability (165 mm s^−1^, 318 g m^−2^ h^−1^). Machine learning techniques were employed to analyze the periodic and dynamic signals of limb swing, achieving a recognition rate of 96.7%. Furthermore, a specialized assisted rehabilitation exercise system has been created to track patients’ rehabilitation progress, monitor their condition, and offer prompt guidance for rehabilitation training.

Research has demonstrated that the lifetime prevalence of lower back pain among athletes varies between 1 and 94%, with the most significant rates noted in rowing and cross-country skiing. The point prevalence of lower back pain, characterized as the percentage of athletes experiencing pain at a specific moment, ranges from 18 to 65%. This percentage is lowest in basketball and highest in rowing [102]. The measurement of the knee and hip joint rotation angles is crucial for determining lower limb kinematics. Wearable systems often utilize IMUs, typically placed on the calf and thigh, due to their superior capacity to detect lower limb activity in comparison to wrist or trunk kinematics, and their rapid convergence with physiological signals [103–105]. In lower limb rehabilitation applications that need to use rigid exoskeletons, Gao et al. [106] proposed the use of a 3D-printed hybrid motion-capture and energy harvesting lower limb (MC-EH-HL) system (Fig. 6d). The MC-EH-HL system is implemented by combining a high-efficiency piezoelectric bimorph array generator based on a slider guide for energy harvesting (S-PEG) and a minimalist joint rotational detection in both directions (R-TENG). The R-TENG is a ratchet-based TENG that provides the system’s functionality. A unique slider-guide configuration allows up to 20 piezoelectric bimorphs to be incorporated on one arm, allowing for high output power and quick charging speeds, even with low-frequency movements. An enhanced ratchet-pawl design is used to determine the direction, angle, and velocity of joint rotation, quantifying lower limb movement and defining all moving joints. Kinematic analyses, in addition to joint rotation detection, allow for the estimation of other physical parameters regarding lower limb posture and dynamics. These features make the R-TENG to be used as a wearable tool for abnormal gait detection, kinematic monitoring, and lower limb function assessment for rehabilitation. The demonstrated MC-EH-HL system offers an energy-efficient platform for low-cost monitoring of lower limb movement and estimation of various motion parameters, eliminating the need for additional sensors.

During both training and competitive sporting activities, individuals can sustain a variety of acute and chronic injuries. The distribution of plantar pressure serves as an indicator of body posture control and foot structure/function, making it applicable to gait monitoring, real-time health supervision, and general well-being evaluation [107, 108]. The significance of footwear in sports and the daily human life is considerable. While static comfort is easily perceived by the user, ascertaining dynamic comfort during various sports competitions poses a challenge. As shown in Fig. 6e-i, Yang et al. [109] proposed a design for a smart shoe utilizing a TENG. The in-shoe sensor pad (ISSP) is affixed to the upper lining above the instep, allowing real-time monitor of the stress distribution on the foot’s rear. Each sensor unit of the ISSP is an air-capsule TENG (AC-TENG), incorporating activated charcoal/polyurethane/microsphere array electrodes. The entire ISSP package is produced using perfusion and 3D-printing techniques. Each AC-TENG has a detection range of 7.27 MPa, adequate for monitoring pressure changes during various motions. The system includes a hybrid power supply for wireless transmission, enabling foot information to be displayed on a mobile phone. Accordingly, addressing the necessity for intelligent sports technology, the ISSP, when attached to a shoe’s upper lining, provides a method to monitor data regarding the movement of the shoe and foot, which can be used to inform the training regimens and footwear design for athletes.

The human foot is a critical organ, instrumental in various sporting activities and key to collecting intelligent sports data. Detecting foot posture enables rapid capture of substantial motion data that can accurately reflect the motion status, duration, and health. However, existing wearable sensors for gait analysis possess several shortcomings such as the requirement for additional power sources, low sensitivity, lack of stability, and the need for skilled operators. These drawbacks restrict their application outside clinical environments. Cheng Yeh et al. [110] have developed a self-powered biomimetic TENG sensor for real-time gait analysis and rehabilitation monitoring. The innovative sensor comprises a highly elastic liquid metal encapsulated within a novel biomimetic sharkskin-like structure, as shown in Fig. 6e-ii. This encapsulation promotes a rapid charge transfer process and long-term stability during the continuous contact-detachment cycles with the bionic layer. Moreover, the unique surface topography of the solid triboelectric layer exhibits hydrophobic properties, which prevents the adhesion of liquid metal during sensing. The cost-effectiveness, scalability, and compatibility of this TENG with diverse detection strategies promote the evolution of gait detection and rehabilitation monitoring toward a user-friendly, on-demand, point-of-care model. The proposed sensors are designed to distinguish between muscles at varying states of health and injury, including the iliopsoas, tibialis anterior, calf triceps, and quadriceps. Furthermore, integration with IoT technology allows for real-time monitoring via smart wearable devices, thus demonstrating significant potential in personalized healthcare and sports science/technology.

TENG-based wearable devices are characterized by their lightweight, comfortable, and user-friendly design. They have the ability to monitor an athlete’s physiological parameters and overall performance in real time. By continuously observing the athlete’s posture, muscle activity, joint angle, and other parameters, TENG intelligent sports equipment can evaluate the athlete’s exercise load, which encompasses elements such as intensity and duration of exercise. The real-time monitoring and analysis capabilities of this intelligent equipment allow for the provision of practical exercise advice and timely detection of potential risks associated with sports injuries. This enables the early warnings and corrective advice vital for injury prevention, thereby reducing the incidence of sports injuries and safeguarding athlete health. In the event of a sports injury, TENG intelligent sports equipment can record the injured individual’s sports data, including pre-injury and rehabilitation data. This information holds significant value for medical professionals and rehabilitators, assisting in the assessment of injury severity and the development of targeted rehabilitation programs. Furthermore, the device can offer personalized rehabilitation guidance by analyzing data from the injured area, thereby enabling the provision of appropriate rehabilitation movements and training plans for the injured individual.

Although TENG-based intelligent sports equipment shows potential in the field of sports injury prevention and rehabilitation, its optimal functions and operating efficiency still need to be further elucidated. The relatively high R&D investment and production costs lead to high market prices, which may also affect the popularity and application scope of the product. Therefore, it is still necessary to continuously explore new materials, processes, and preparation methods to improve the cost performance of devices. At the same time, a large amount of personal information collected by TENG’s intelligent sports equipment in the field of sports injury prevention and rehabilitation must be protected from being violated and exploited. For instance, the accuracy and stability of the equipment need to be improved, and the algorithms used for data processing analysis need to be optimized. In addition, how to integrate TENG into smart devices and be compatible with other systems is also a big technical problem. In order to study the prevention and rehabilitation of sports injuries and rehabilitation, it is necessary for the equipment to have the ability to dock with medical equipment and rehabilitation systems, and to realize data sharing and collaborative work. With the development of personal medical monitoring and wearable sensing technology, these highly sensitive, mass-produced, and long-lasting TENG-based devices will have a disruptive impact on personal medical monitoring and become the basis for the development of advanced wearable sensing devices.

## TENG Data Analysis Utilizing AI/ML Techniques for Smart Sports

**Fig. 7:**
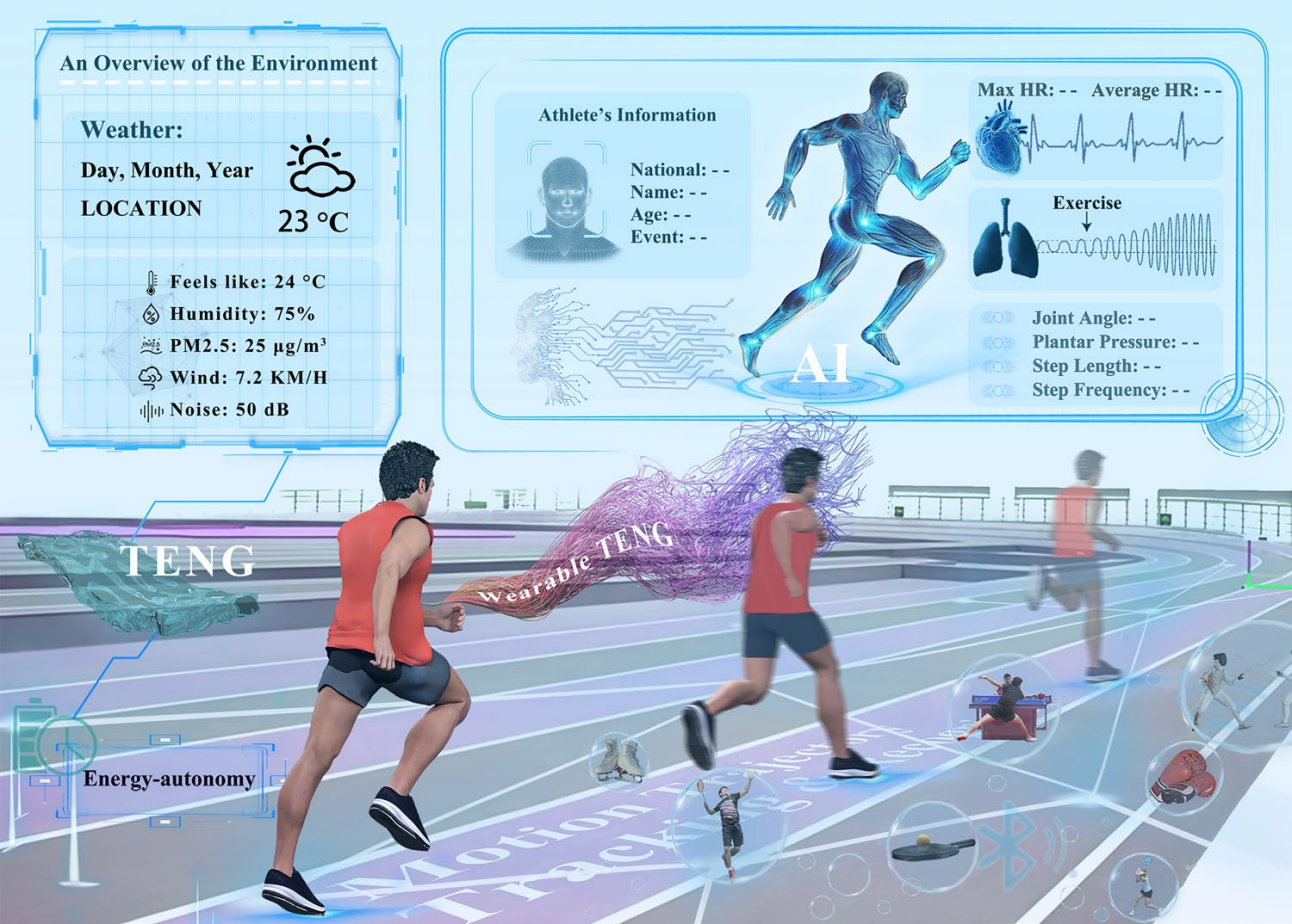
The combination of TENG and AI/ML boosted smart sports

Following Industry 4.0 and Health 4.0, Sports 4.0 has gradually emerged. As the core driving force of the fourth industrial revolution, artificial intelligence (AI) is developing toward more application scenarios. It is increasingly evident AI and big data will play a more important role in sports [111]. The International Olympic Committee’s formal announcement of the ‘Olympic AI Agenda’ indicates the further integration of sports and AI. Human society is moving rapidly toward a new era of ‘AI + sports.’ Machine learning (ML), a fundamental component of artificial intelligence (AI), serves as an indispensable tool for advancing AI capabilities. This computational paradigm enables systems to autonomously improve performance through data-driven pattern recognition and iterative optimization [112–114]. Based on computer vision and deep learning technology, AI can automatically track the targets and collect and analyze athletes and venues through wearable devices, sensors, cameras, and other photographic apparatus, thus changing digital sports and intelligent sports. The integration of ML technology with TENGs enables real-time data monitoring while simultaneously delivering personalized training recommendations to athletes, thereby advancing the development of intelligent sports systems. Notably, TENG, as an innovative self-powered sensing device, has taken intelligent sports to a new height in many aspects (Fig. 7).

AI technology has significantly contributed to the construction of sports event venues and their associated facilities. For instance, sensors were employed at the FIFA World Cup Qatar 2022 to measure humidity and temperature conditions, which were then adjusted using intelligent air-conditioning and other systems. This ensured that the temperature, humidity, sunlight, and other factors were optimal for diverse regions within the stadiums, thereby enabling athletes to better adapt to the competition environment [115, 116]. Analogously, the Paris 2024 Olympic Games utilized AI monitoring technology to protect athletes’ network security and prevent cyberattacks and harassments [117, 118]. TENG, an innovative energy and sensing technology, offers unique advantages in its integration with AI technology. It can function not only as a sensing terminal for accurate monitoring of environmental parameters but also as an energy source for efficient conversion of mechanical energy in the environment into electric energy [119–121]. This dual-technology approach, integrating energy supply and environmental monitoring, has shown immense potential in the sports field. It has not only optimized the processes of sports training and competitions but also introduced new momentum into the intelligent evolution of the sports field, marking a new era in sports.

Wearable devices offer significant potential for AI research. Their ability to collect and monitor data, coupled with their capacity for real-time feedback, fosters the development of big data and behavioral modification [122, 123]. By gathering a substantial quantity of an athlete’s physical data during daily training and performance data during competitions, and conducting real-time analysis of key data points, these devices can provide invaluable decision-making support for coaches. This enhances the relevance, scientific rigor, and effectiveness of training regimens and enables coaches to adjust tactical strategies instantaneously, thereby improving overall competition performance. For example, TENG can be integrated into the insoles of running shoes for monitoring pressure distribution and stride frequency during running. By analyzing this data through machine learning, athletes can adjust their running posture and improve their overall athletic performance. Liu et al. [124] developed a four-layer friction-enhanced TENG based on loofah-conducting graphite (LG-TENG). By integrating the LG-TENG into athletic insoles and elastic straps, athletes’ exercise data (e.g., stride frequency, stride length, and muscle contraction status) can be monitored in real time. The study demonstrated the output signals of LG-TENG at different running speeds, which can accurately analyze the gait and movement patterns of athletes. LG-TENG can be used to analyze athletes’ deep squatting and jumping movements, and to assess athletic performance and explosiveness by monitoring the state of muscle contraction. This study uses deep learning models, such as medium neural network (MNN) and long short-term memory (LSTM) network, to classify and recognize seven movement speeds by collecting plantar triboelectric signals at different movement speeds. The findings demonstrate that the MNN model attains 98.1% accuracy in recognizing seven speeds for a single individual, while the LSTM model achieves 96.46% accuracy in recognizing seven speeds for a triple person. Furthermore, TENG can be integrated into sports equipment (e.g., ski poles, rackets, and bicycle handlebars) to assess the condition of the equipment and the performance of athletes. Yuan et al. [125] presented a novel self-powered intelligent badminton racket (SIBR) that operates through the utilization of the triboelectric and piezoelectric effect. The SIBR involves the deployment of a triboelectric sensor array, which is formed by the spraying of conductive silver paint onto the strings of the badminton racket. When a badminton ball comes into contact with the strings, the triboelectric effect generates the electrical signal that is used to monitor the stroke position. The TENG sensor array enables the system to monitor the position of the badminton stroke in real time and recognize whether the stroke point is in the optimal area. The precise monitoring of the stroke position enables the player to adjust their stroke action, thereby enhancing the quality of their stroke. The classification of electrical signals of the hitting position by a LSTM network has been demonstrated to achieve a classification accuracy of 95% in identifying the hitting points in six different regions. ML algorithms have been employed to transform complex electrical signals into intuitive feedback of the hitting position, thus assisting athletes in optimizing their hitting technique. This self-powered sensing system has the potential to enhance the scientific and efficient aspects of training, as well as providing a foundation for the development of intelligent sports training systems.

Disputes regarding the fairness of referees’ scoring and penalty decisions are not uncommon. The incorporation of AI, distinguished by its comprehensive, astute, objective, and impartial nature, can significantly enhance the accuracy and fairness of refereeing. At present, AI-assisted refereeing is emerging as a new trend in sports officiating. However, the widespread use of intelligent devices presents an energy consumption issue. As a sustainable energy source and distributed sensing terminal, TENG embodies the concept of ‘killing two birds with one stone’ [126, 127]. For example, Chen et al. [128] developed an elastic piezoelectric-triboelectric hybrid yarn (E-PT yarn) with a micro-nanostructure on its surface capable of generating triboelectric and piezoelectric signals simultaneously. The E-PT yarn was woven into a 9-channel sensor array integrated into the strings of a tennis racket. When a tennis ball hits the racket, the sensor array detects the force and position of the impact and generates corresponding electrical signals. These signals are analyzed by ML algorithms to help the player optimize the angle and position of the shot. The electrical signals from elbow movements are classified by a convolutional neural network (CNN) to recognize six common tennis postures (e.g., forehand, backhand, serve) with 95% classification accuracy. The machine learning algorithm is able to translate complex electrical signals into intuitive postural feedback to help athletes improve their technical movements. Combined with the system's ability to determine whether a shot is over the net or out of bounds, it provides real-time feedback and helps athletes improve their shot accuracy. In addition, as a self-powered sensor, TENG has shown significant potential in confrontational sports (e.g., martial arts characterized by fast and high impact). Traditional refereeing decisions are prone to error, which are difficult to quantify the training effect on athletes. To address these issues, Hassan Ahmadi et al. [129] designed a flexible and lightweight TENG (FL-TENG) and used it in martial arts training and protective equipment. FL-TENG can detect the force and location of an athlete’s punch, recognizing an ineffective punch (e.g., an underpowered punch) or a foul punch (e.g., hitting a no-go zone). By integrating multiple TENG sensors into training equipment (e.g., helmets, punching bags), a comprehensive data set can be generated for analyzing an athlete’s performance and detect foul strikes. When an athlete hits a forbidden area (e.g., groin, forearm), the TENG generates a corresponding voltage signal, and if a set threshold is exceeded, a foul is detected. The researcher used various classifiers, such as K-nearest neighbor (KNN), support vector machine (SVM), logistic regression, and others, to train the model and evaluated the performance of the model using a confusion matrix. The results showed that the KNN model performed best on the non-linear dataset with an accuracy of 93.75%.

In high-intensity training and competitive environments, injuries and illnesses are inevitable aspects of an athlete’s career. Balancing performance with injury prevention is of paramount importance in sports. By analyzing athletes’ performance and injury data in real-time, AI can detect potential injury risks, assist in formulating reasonable training plans, and prevent injuries. Post-injury, AI can aid in developing treatment and rehabilitation plans, facilitating the athlete’s return to competition through data collection and analysis. AI technology holds significant potential in injury prediction, performance analysis, personalized training, and treatment. However, it faces challenges related to the dynamic complexity of sports and the multidimensionality of athletes’ performance [130]. The amalgamation of TENG and AI technologies presents a novel opportunity for the intelligent development of sports. This integration can not only address the challenges faced by AI in handling complex sports data but also provide athletes with more comprehensive and accurate support in training and competition. Using the movement data collected by TENG, ML algorithms analyze athletes’ movement patterns, identify technical deficiencies and provide recommendations for improvement. Liu et al. [131] introduced a green and recyclable bamboo paper-based TENG (BP-TENG) based on the traditional Chinese papermaking process and demonstrated its application in self-powered sensing and intelligent detection systems. The application of BP-TENG in long jump pedaling can help athletes optimize their training performance by monitoring their pedaling movements to predict the jump distance. After BP-TENG collected the pedaling data from the athletes, the data was pre-processed and classified using ML algorithms. A variety of ML algorithms (e.g., decision tree, naive Bayes, SVM) were used in the study and the SVM algorithm was finally selected for the classification and prediction of jump signals, with an identification accuracy of up to 99.8%, which is capable of providing personalized training suggestions for athletes. BP-TENG can monitor athletes’ movement posture and strength in real time, detect potential risks of sports injuries in time, and provide early warnings to help athletes adjust their movements to reduce the probability of injury. Similarly, Feng et al. [132] introduced a biodegradable and environmentally friendly TENG based on sodium alginate and demonstrated its application in self-powered sensing and real-time sports injury monitoring. The application of this TENG in Taijiquan real-time monitoring of knee movement during exercise accurately detects correct half-squat posture and incorrect posture, thus preventing knee injuries. When combined with advanced narrow neural network (NNN) technique, the TENG is capable of analyzing knee motion data with an accuracy of 97.96%, thus providing valuable data support for the treatment and rehabilitation of knee osteoarthritis.

Powered by advancements in AI technology, TENG technology has been widely applied across various fields such as smart homes, intelligent ambient environments, transportation systems, and smart medical care [133–137]. These implementations have laid a robust groundwork for the ongoing amalgamation of TENG and AI technology within the sports industry. Currently, the integration of AI technologies such as ML and deep learning (DL) with TENG technology in sports primarily focuses on human data acquisition, processing, and classification tasks. For instance, TENG sensors collect kinematic data (e.g., gait, cycling postures, and muscle activity), which are subsequently classified by ML or DL algorithms (e.g., SVM, random forest, or CNN) to achieve functionalities like motion pattern recognition, abnormal movement detection, or fatigue state analysis. However, such implementations exhibit inherent limitations: systems often remain passive and static, merely classifying and analyzing acquired data without real-time predictive feedback, dynamic adaptability, or generalizability optimization during athletic processes. Furthermore, application scenarios remain relatively limited, failing to fully exploit ML/DL’s potential in personalized training optimization, sports injury prediction, and intelligent interaction.

To overcome current limitations, future research could explore advanced algorithms and application directions. For example, spiking neural network (SNN) could simulate neuronal firing patterns to enable real-time motion optimization and enhance users’ performance by dynamically adjusting training intensity or posture. Time-series prediction models (e.g., LSTM or Transformer) might forecast sports injury risks or health status evolution, delivering preventive recommendations. Integrating federated learning with differential privacy could facilitate multi-device collaborative training while preserving user privacy. Multimodal data fusion techniques could combine TENG sensors with other sensors (e.g., accelerometers, heart rate belts) to improve monitoring comprehensiveness and accuracy. Additionally, lightweight models (e.g., MobileNet, TinyML) and edge computing could address real-time processing and computing resources constraints, enabling efficient application of DL/ML and TENG technology in smart sports equipment and feedback platforms. These integrated technology with algorithms may drive deeper DL/ML and TENG convergence, potentially enabling intelligent, personalized, and efficient athletic experiences in sports field.

## Summary and Prospective

**Fig. 8:**
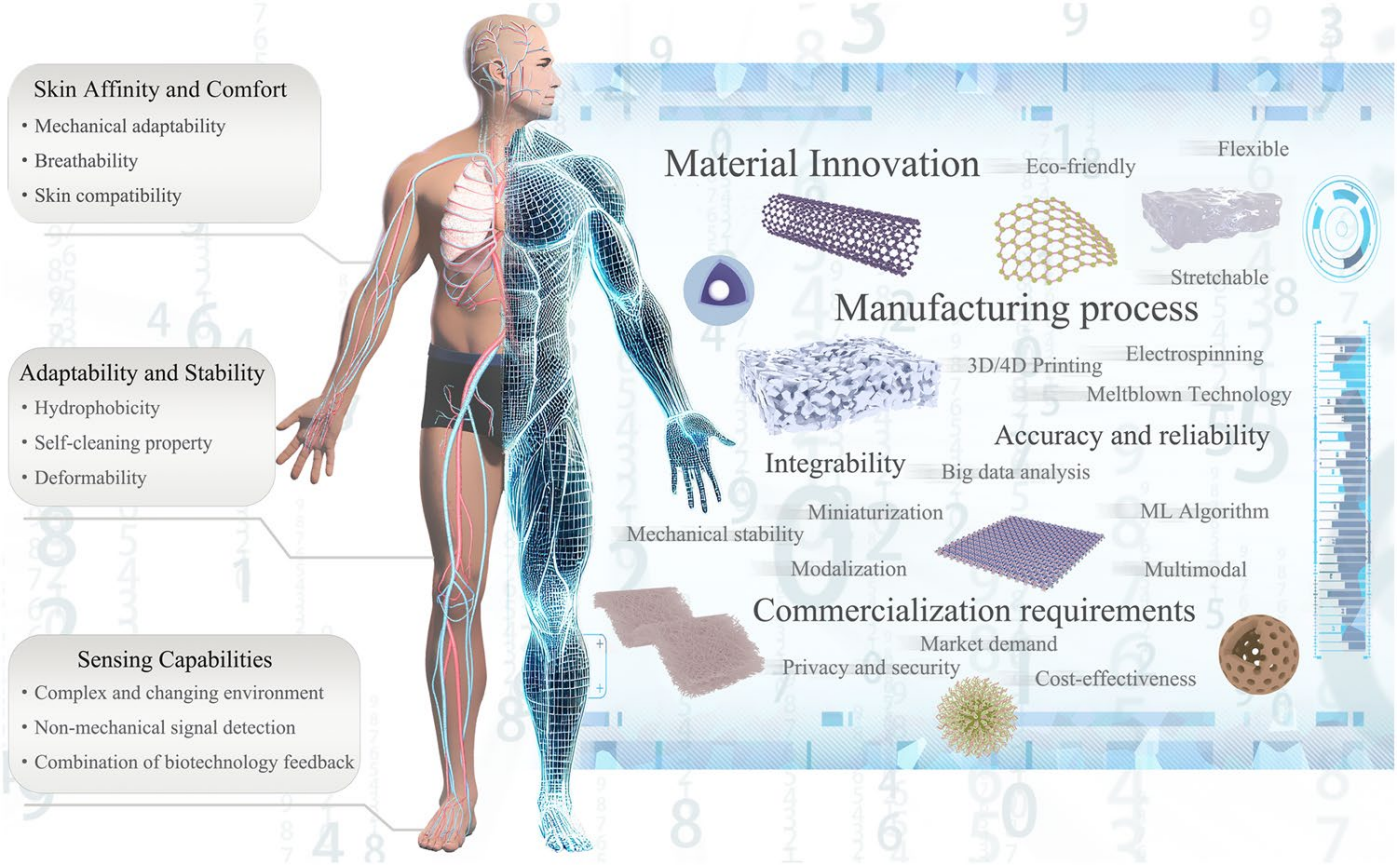
Outlook on self-powered intelligent sports research

This paper provides an exhaustive overview of the fabrication and utilization of intelligent sports equipment based on TENG, emphasizing four critical aspects: the monitoring of sports signals, the facilitation of sports training, the provision of event refereeing services, and the prevention/rehabilitation of sports injuries. First, TENG’s unique electromechanical conversion characteristics allow it to generate different electrical signals in response to external stimuli, eliminating the need for an external power source. This enables real-time, self-powered monitoring of pulse/heartbeat/breathing, body motions, and other related physiological information. Second, TENG’s exceptional ability to harvest low-frequency mechanical energy gives it a distinct advantage in building intelligent motion-sensing networks. When integrated with other sensors or circuits, TENG can form hybrid systems with enhanced functionality. Additionally, due to its high-voltage, low-current output characteristics, TENG is highly biocompatible for human body use. The comfort and skin affinity of athletes can be ensured through the careful selection of materials and appropriate structural design. Table 2 compares commercial wearable devices with those based on TENG in the context of smart sports. Although TENG-based intelligent sports equipment has significantly advanced smart sports, there are still challenges. Areas for potential improvement in TENG-based intelligent sports monitoring equipment include material innovation, fabrication process, skin affinity and comfort, adaptability and stability, sensing capability, accuracy and reliability, and commercialization (Fig. 8). Increased attention to these areas could enhance the equipment’s applicability in future research.

1. *Material innovation* It is well established that the vast majority of materials exhibit triboelectrification effects, ranging from wood and polymers to metals and even silk [178, 179]. For TENG to be more suitable for power supply applications, higher output power and energy conversion efficiency are essential across a broad spectrum of stable applications. The incorporation of innovative materials and the investigation of micro-nanostructures can bolster the electrical properties and durability of friction materials. Notably, synthetic polymers provide enhanced durability, strength, and versatility [180, 181]. Yet, their non-biodegradable nature poses significant environmental challenges. Consequently, there has been a burgeoning interest in biocompatible alternatives to mitigate environmental concerns. Biopolymers from renewable sources present an eco-friendly alternative, though they often suffer from shortcomings such as limited durability, high water vapor permeability, brittleness, low heat resistance, degradability, and reduced processability [182]. Crafting materials that are breathable, flexible, eco-friendly, and stretchable materials (e.g., super flexible wood) represents a significant challenge in meeting the rigorous requirements of wearable applications, including e-skins, smart patches, and textiles. To achieve biodegradability, future TENGs might explore strategies like natural polymer modification and the utilization of natural/synthetic composites, enhancing material adaptability to various decomposition media.
2. *Fabrication process* Being a new type of sensor, there is currently no standardized fabrication process for TENG. The lack of standardized processes and the extensive manual labor in laboratories is a major challenge toward large-scale implementation of this technology. The traditional manufacturing methods cannot meet the requirements of personalized spatial structure and high triboelectrification performance of TENG [183, 184]. Although some fabrication methods of TENG devices, such as electrostatic spinning, photolithography, and 3D-printing [185, 186], have been reported, most of the methods are featured with high cost, long processing period, and high complexity. For instance, the development of 3D/4D-printing technology has allowed the manufacture of precision and personalized sensors to better adapt to irregular body shapes, but the limitation of materials available on the market highlights the need for further research and the exploration of more materials. Nanofibers prepared by electrostatic spinning have an ideal surface area-to-volume ratio, large contact surface area, and laminated porous structure [187]. However, low efficiency, high energy consumption, safety issues, unstable fiber bonding, and charge distribution are the main limitations of this preparation method. Therefore, exploring a fabrication process that is convenient to operate and low in cost is a good choice for commercializing TENG in intelligent sports, which will be beneficial for its all-in-one integration into different application scenarios. Melt-blown is a widely used technology for the production of non-woven fabrics with large specific surface area. It provides a solvent-free, large-scale, low-cost, and recyclable fabrication process suitable for industrialization [188].
3. *Skin affinity and comfort* Traditional wearable sensors suffer from limitations such as rigidity, bulkiness, and the need for recharging. Consequently, it is crucial to explore and develop intelligent wearable devices that do not hinder sports performance [189, 190]. Most smart wearable TENGs for motion monitoring are in intimate contact with human skin. High skin affinity and mechanical compliance are required for best comfort. Fiber/textile materials provide a better option than film-based TENG technology, which can provide enhanced triboelectrification while processing intrinsic breathability and high mechanical properties for adapting to human motion and allowing quick evaporation of sweat and other metabolites. By innovative material selection and optimized structural design, the dermal compatibility and wearing comfort of wearable TENGs can be significantly improved, which will broaden their applications in complex body motion monitoring. Breathable, highly flexible, high-power-density electronic skin sensors via low-cost preparation processes are worth exploring. Meanwhile, intelligent fibers present a promising solution, not only serving as the ‘raw materials’ for smart apparel, but also finding applications in sports equipment. They facilitate motion sensing and data collection without impacting athletic performance [191–193]. The TENG-based intelligent fiber combines traditional fiber functions such as heat retention, breathability, and moisture absorption with advanced TENG technology. This amalgamation allows it to sense environmental changes and generate corresponding responses, enhancing its intelligent attributes. The fiber offers benefits including energy harvesting, self-powering, environmental friendliness, high performance and compatibility, flexibility and comfort, multifunctionality and integration, as well as durability and stability. These advantages grant the TENG-based intelligent fiber widespread application prospects and immense market potential in the sports industry [194–197]. Furthermore, this technology resolves the issue of frequent recharging required by traditional wearable devices, improving device portability and aligning with sustainable development trends in sports. Concurrently, its intelligent sensing and data analysis capabilities provide athletes with personalized training guidance and feedback.
4. *Adaptability and stability* For a self-powered sensor, it is imperative for the TENG accommodating the intricate dynamics of human motion, thereby minimizing signal disruption and bolstering sensing accuracy. Enhancing the material’s deformability and reducing device thickness are pivotal steps to amplify the mechanical compliance and response sensitivity of wearable TENGs. These devices should not only align with human body motions but also resist dynamic disturbances, facilitating effective energy conversion and precise physiological monitoring. The TENG’s stable functioning can be affected by various human body movements, influenced by sweat, body fluids, and other multifaceted physicochemical properties. As such, there’s an imperative for TENGs to transition from static to dynamic devices that can proactively adapt to environmental shifts. This adaptation is realized through modifications in the device’s material properties and microstructure. The hydrophobic and self-cleaning attributes of the triboelectric layer can be augmented by designing surface nanostructures or introducing hydrophobic chemical groups to the surface. Additionally, advanced encapsulation methods can be devised to protect TENG devices from sweat intrusion while preserving their electrical integrity. Notably, most existing devices are prepared using molds, which significantly increases device thickness. Exploring TENG devices with diverse material microstructures, integrated functionalities, and transient operational states is essential to guarantee their dependable performance across diverse conditions.
5. *Sensing capability* The term ‘sports load’ is commonly segmented into two interrelated categories: internal load and external load. ‘Internal load’ quantifies the psychophysiological response of the athlete to the external load during the exercise process. It can be measured via various physiological indicators such as heart rate, blood lactate, blood pressure, blood oxygen saturation, blood glucose, and urinary protein. These indices are instrumental in evaluating the athlete’s physical condition and fatigue level. Conversely, the ‘external load’ quantifies the work executed by the athlete. The primary goal of sports performance monitoring is to appraise the efficacy of an athlete’s training program by examining their training performance. Athletes’ training levels can be determined by observing parameters like speed, strength, and endurance, among others. In many sports, the key to optimizing the training program and enhancing performance hinge on studying the relationship between the athlete’s internal and external exercise loads. However, the current level of TENG technology might fall short of fully satisfying this requirement. Although TENG can function as an active sensor to monitor mechanical signals, detecting non-mechanical signals such as sweat and blood composition during sporting activities presents a challenge. In particular, real-time monitoring and analysis of data like sweat composition, electrolytes, EEG, EMG, etc., are underdeveloped in complex and variable sports environments. Future research integrating smart devices based on TENG with combined biofeedback technology may provide more precise guidance for athletes in rehabilitation training. For instance, muscle contraction can be evaluated through the observation of muscle electrical activity, allowing for the real-time adjustment of training intensity and mode.
6. *Accuracy and reliability* Intelligent motion equipment employing TENG must ensure the requisite accuracy and stability when assessing motion parameters, as this is vital for the precision and reliability of the data collected. Nevertheless, current technological advancements might fall short in meeting these standards, especially in dynamic and complex sporting environments. The accuracy of wearable devices can be augmented by integrating various transducer types or by implementing multimodal or multiplexed sensing on a unified platform. This allows for the simultaneous measurement of diverse analytes or samples. Most TENG-based transducers employed in motion monitoring rely on basic waveform or amplitude of output voltages to discern different motions [198–200]. This method overlooks the intricate correlation between multiparameter signals from multiple transducers and the unique motion patterns of the human body. Incorporating machine learning into motion data analysis can optimize the use of information collected by TENG-based sensors, offering a more precise depiction of athletic performance. Such devices have the potential to offer athletes highly personalized training insights by leveraging a thorough analysis of their sports data via machine learning algorithms.
7. *Integrability* The practical utilization of wearable TENGs demands their miniaturization and modularity to ensure smooth integration with the human body, clothing, or machinery. The construction of a wireless sensor network using TENG technology requires several components, such as data processing units and wireless transmitters and receivers, to establish a highly modular sensing system. In the context of thin-film-based TENG, the design of self-adhesive materials is an effective strategy to enhance their integration with the substrate. For textile-based TENG integration, the utilization, traditional textile engineering techniques like knitting, sewing, and weaving offer promising avenues for exploration. The primary challenge lies in enhancing the mechanical properties of fiber/textile-based TENG, particularly striking a balance between their mechanical stability and electrical fidelity.
8. *Commercialization requirements* The future market potential of TENG technology in the intelligent sports sector, among various energy harvesting and sensing technologies, depends on its market demand and cost-effectiveness. To increase the applicability and utility of intelligent sports equipment, the range of materials used to produce TENG must be expanded. For instance, using the same materials in TENGs as those used in sports facilities or wearable devices is a promising strategy. There is also a need to design personalized TENG-based smart equipment for specific sports to meet market requirements. To boost the market competitiveness of TENG-based smart equipment, it may be crucial to optimize the production process and supply chain management further, focusing on enhancing production efficiency, lowering production costs, and improving performance. However, gaining the social acceptance of TENG-based intelligent sports monitoring gear is crucial. This can be accomplished by informing users of the gear’s benefits and drawbacks, as well as its integration with existing wearables and healthcare services. The gear should also be integrated into IoT applications that include data security and processing protocols. The establishment of an ethical regulatory framework for wearable data networks could also be helpful in encouraging usage.

**Table 2:** Comparison of commercial and TENG-based smart wearable equipment in intelligent sports

	Commercial sports wearable detection equipment	Sports wearable detection equipment based on TENG
Energy supply	Battery power needs to be recharged repeatedly [138]	No need to charge, self-powered [139]
Fabrication	Mass production, high degree of device standardization [140]	Simple to fabricate [141], manually assembled [142]
Weight and volume	Customizable size and volume [143], heavy [144]	Small size, volume and weight [145]
Comfort	Uncomfortable if worn for a long time, wearer is easily distracted [146]	Imperceptible to the wearer [147], breathable [148], stretchable [149]
Function	Heart rate [150], acceleration [151], distance, Frequency [152], positioning [153], rotation [154], angle, load [155], blood pressure, blood oxygen [156]	Heart rate [157], blood pressure, frequency [158], amplitude, acceleration [159], sweat detection [160]
Signal	High precision, good stability [161], not easily affected by sweat and temperature [162], fast unlimited transmission rate [163]	Greatly affected by the external environment [164], low accuracy [165]
Characteristic	Strong and durable [166]	Antibacterial [167], recyclable [168], self-Degradable [169], conformable [170]
System integration	Multisensor integration and higher integration [171], good data wireless transmission performance [172], good software and hardware compatibility [140]	Low integration, single sensor type [173], poor wireless transmission performance [149]
Data detection and analysis	Beautiful interface, real-time and accurate collection of statistical data, data cannot be predicted, cumulative errors will form over time [174], large sampling rate range [175], trajectory analysis [176], risk of personal data leakage [177]	Collect statistical data in real-time and be able to predict and judge results, no secondary processing of data is required [145]
